# Network hub-node prioritization of gene regulation with intra-network association

**DOI:** 10.1186/s12859-020-3444-7

**Published:** 2020-03-12

**Authors:** Hung-Ching Chang, Chiao-Pei Chu, Shu-Ju Lin, Chuhsing Kate Hsiao

**Affiliations:** 1 Division of Biostatistics, Institute of Epidemiology and Preventive Medicine, National Taiwan University, No. 17, Xu-Zhou Road, Taipei, 10055 Taiwan; 2 Institute of Statistical Science, Academia Sinica, Taipei, 11529 Taiwan; 3 Bioinformatics and Biostatistics Core, Center of Genomic Medicine, National Taiwan University, Taipei, 10055 Taiwan

**Keywords:** Direction regularization, Intra-network, Neighbor correlation, Pathway activity score, Topology measure

## Abstract

**Background:**

To identify and prioritize the influential hub genes in a gene-set or biological pathway, most analyses rely on calculation of marginal effects or tests of statistical significance. These procedures may be inappropriate since hub nodes are common connection points and therefore may interact with other nodes more often than non-hub nodes do. Such dependence among gene nodes can be conjectured based on the topology of the pathway network or the correlation between them.

**Results:**

Here we develop a pathway activity score incorporating the marginal (local) effects of gene nodes as well as intra-network affinity measures. This score summarizes the expression levels in a gene-set/pathway for each sample, with weights on local and network information, respectively. The score is next used to examine the impact of each node through a leave-one-out evaluation. To illustrate the procedure, two cancer studies, one involving RNA-Seq from breast cancer patients with high-grade ductal carcinoma in situ and one microarray expression data from ovarian cancer patients, are used to assess the performance of the procedure, and to compare with existing methods, both ones that do and do not take into consideration correlation and network information. The hub nodes identified by the proposed procedure in the two cancer studies are known influential genes; some have been included in standard treatments and some are currently considered in clinical trials for target therapy. The results from simulation studies show that when marginal effects are mild or weak, the proposed procedure can still identify causal nodes, whereas methods relying only on marginal effect size cannot.

**Conclusions:**

The NetworkHub procedure proposed in this research can effectively utilize the network information in combination with local effects derived from marker values, and provide a useful and complementary list of recommendations for prioritizing causal hubs.

## Background

In a disease-associated biological pathway containing nodes such as genes, proteins and other chemical compounds, the detection of nodes that are crucial to this pathway activity may help elucidate the molecular mechanism influencing the response of interest. The prioritization of nodes in this association pathway may provide useful information for follow-up experimental validation and thereby facilitate the search for drug target molecules [1, 2]. In the medical genetics community in recent decades, the prioritization of a set of genes has been an important issue, especially when the research focus is on identification of genes for drug discovery [3, 4].

Current methodology for such prioritization can be grouped into three categories, depending on what information is utilized in the procedures of modeling and ranking. The first group is more traditional; these methods rank the genes in a previously identified gene-set or pathway based only on marginal marker values, such as the fold-change, the t-statistic, or a function of the t-statistic, if gene expression data are involved. These methods are straightforward to carry out and can quite effectively reduce the enormous number of genetic markers to one that is easier to handle. However, these methods may not be proper with the underlying assumption that these genes are independent, as noted in many gene-set analyses [5–9]. Through correlation sharing or through de-correlation such as in the shrinkage correlation-adjusted-t (shrinkage cat) score [10], several modifications to this group of tests have been proposed. Another inappropriate assumption is that of exchangeability, which assumes all candidate genes are equally important a priori, regardless of whether they are hub genes or not. This assumption ignores the fact that some genes, such as hubs, interact with relatively more other genes.

Unlike the above methods which use only expression data in their prioritization analysis, the second group of approaches utilizes information from networks that are established based on research findings from the literature and data from multiple sources to define *seed genes*, calculates similarities between the seed and each of the candidate genes, and then ranks the candidate genes based on the similarities. This concept is usually called guilt-by-association. Examples include Endeavour, ToppGene and GeneDistiller [11–13]. These methods depend heavily on the information available and would be helpful for investigating reproducibility. However, they assume the network to be static, which may inflate false positive results, and some of them do not account for the marginal effects [14].

The third group of analyses integrates both the gene expression data regarding the phenotype of interest and the network information. For instance, PINTA [15, 16] prioritizes candidate genes by combining the protein association network from STRING [17], the disease expression data, and a kernel for distance. The network gene prioritizer (NGP) proposed by Wu et al. [14] uses the correlation under the network rewiring (NR) model or networked differential expression (ND) model to construct a network for each candidate gene, and then carries out gene set enrichment analysis (GSEA) [18] to determine the importance of the genes, called NGP-NR and NGP-ND respectively. These approaches incorporate the network information through correlations. In contrast, with pathways defined in KEGG [19], Lin et al. [20] included expression levels, correlations and degree of the nodes in their Bayesian probabilistic prioritization procedure. The degree they included in their method provides the information about the pathway/network structure and topology.

When a pathway is represented as a network, the structure may provide useful information. For instance, in the network plot, any two nodes having a direct molecular interaction, whether inhibitory or activating, are immediate neighbors of each other; while nodes that have not been found to interact will not be directly connected in the pathway network. A node with a large number of neighbors is considered as a hub gene node. A hub may be more crucial than other nodes, since its absence can disrupt the pathway function and isolate other gene nodes [14, 21–25]. The number of neighboring nodes of a gene is called its degree, which can be derived from the adjacency matrix of the network [26]. In other words, the observed degree distribution or the adjacency matrix of this network can provide a description of the structure of the pathway. Such an affinity measure for the network topology may offer useful information in ranking gene nodes and in summarizing the collective enrichment of a pathway. Similar ideas have been adopted in testing the association of a network/pathway, but not in ranking gene nodes [27–29]. Although these topology-based methods include information about the pathway structure, such an affinity measure has not been utilized in identifying influential hub gene nodes.

**Fig. 1:**
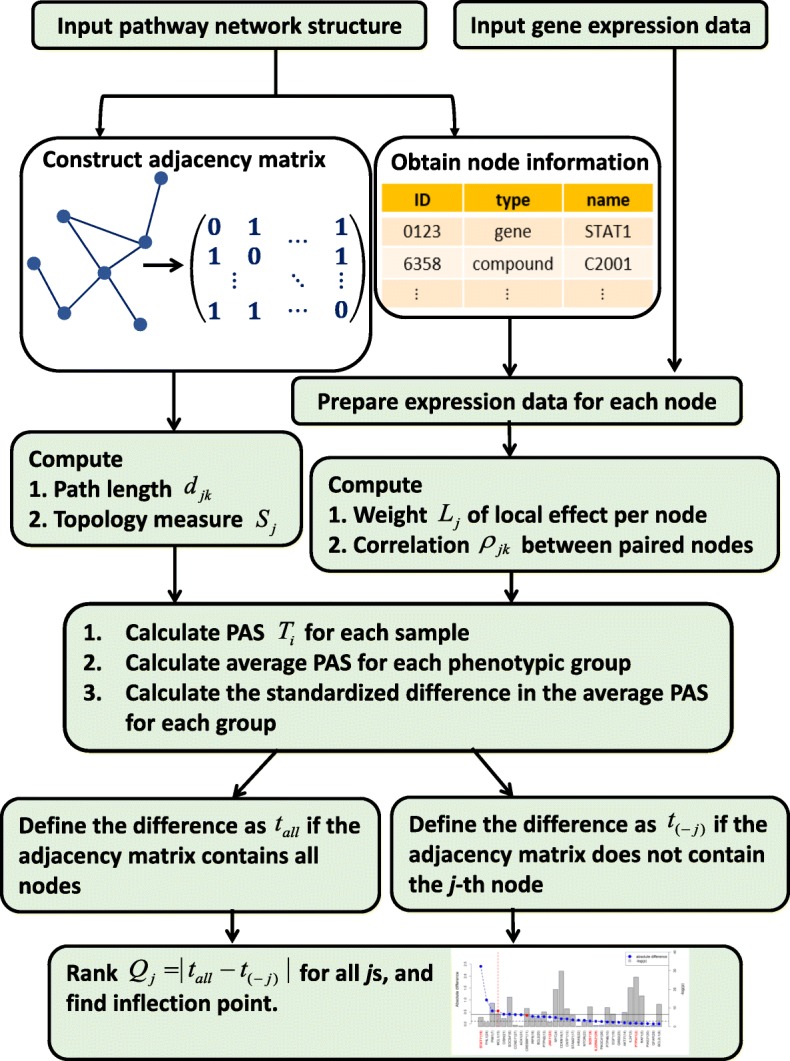
Flowchart of the NetworkHub analysis

In this analysis, we propose to construct the intra-network information that each node carries in the pathway, calculate the local effect from the marginal influence, weight the local and network information separately, and combine them to formulate a pathway activity score. The intra-network information is composed of the pairwise affinity measures of correlation and distance. Based on the adjacency matrix of the network, the shortest distance between two nodes is called the path length, where the length can represent the efficiency of information transmission in this network. The resulting pathway activity score for each sample is next used to prioritize the gene nodes, particularly the hub nodes. We call the method the NetworkHub procedure (Fig. 1).

The rest of the article is organized as follows. The methodology and the underlying rationale will be explained in the Methods section. In Results, applications and simulation studies are conducted to evaluate the performance of the proposed procedure and to compare it with other existing methods, including ones that prioritize with and without correlation, such as the shrinkage cat and t tests, and ones that prioritize with and without network information, such as Endeavour, PINTA, NGP, and Lin’s method. We then conclude with the Discussion section.

## Results

### Breast cancer with DCIS (RNA-Seq)

The first application considered for illustration is a study of high-grade ductal carcinoma in situ (DCIS), a subtype of breast cancer [30]. This study collected RNA-Seq data from 10 unaffected subjects and 25 breast cancer patients, which can be downloaded from the NCBI GEO database (accession number GSE69240). The downloadable data set was examined with procedures for quality control and normalization, as described in the original report of the study [30]. Four pathways defined in KEGG [19], P53, mTor, Estrogen, and JAK-STAT, are selected here as networks for demonstration. These pathways have been reported to associate with breast cancer [31], and have passed the global test [32], GSEA [18], Fisher’s test, SPIA [33] and the Bayesian association test in Lin et al. [20]. For each pathway, the plots of *L*_*j*_ and *S*_*j*_ against path length in Figure S1 show that the local weight of each node neither associates with nor reflects the magnitude of its degree, whereas the topology weight does increase slightly with the degree of the node. The NetworkHub procedure is used to rank the gene nodes inside each pathway network, respectively, with the leave-one-out evaluation.

**Fig. 2:**
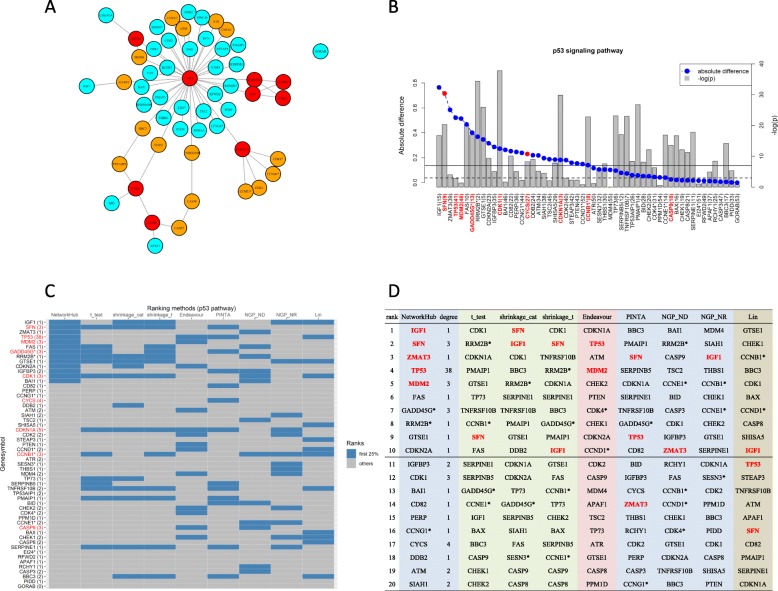
P53 pathway of the breast cancer study. **a** Network plot. Nodes in red are hubs with degree ≥3, nodes in yellow are of degree 2, and nodes in light blue are of degree ≤1. **b** Scree plot (blue dots) of the relative influence of gene nodes (left Y-axis). The right Y-axis is the negative-logarithm-*p*-value (grey bar). The dashed red line corresponds to *p* = 0.05, and the solid red line to 0.05 divided by the number of nodes inside the pathway. The X-axis label indicates the gene node. A name followed by an * indicates more than one gene is contained inside this node. The number in parentheses is the ranking determined by *p*-value. **c** Heatmap for comparing rankings under different methods. Gene nodes ranked among the first 25% are colored in grey. The nodes considered hubs are denoted in red. The number in parentheses after the gene symbol indicates the degree of the node. **d** Ranking list

For the P53 pathway, its network plot is shown in Fig. 2a. In the scree plot in Fig. 2b, the gene nodes on the X-axis are ordered according to their importance with bold red font used to indicate hub nodes, while the grey bars represent the magnitude of negative log-*p*-values. Several interesting findings are indicated when the NetworkHub procedure is implemented. First, note that the top-ranking node *IGF1* in this pathway indeed is known to have crosstalk with estrogens; anti-estrogens such as tamoxifen have served as a routine treatment for breast cancer in many countries [34]. The use of IGF1R inhibitors as molecule targets has been considered in several recent clinical trials, including a phase Ib/II trial (NCT02123823) of the drug BI 836845 and a phase II study of BMS-754807 combined with letrozole (NCT01225172). More reviews can be found in [34]. This top-ranking gene node has a significant marginal effect and therefore it is not surprising that it has been included in several clinical investigations. This gene node is, however, overlooked when some other methods are applied. For instance, as seen in Fig. 2c, *IGF1* is not in the top 25% under Endeavour, PINTA, NGP-ND and NGP-NR; but it ranks second under the shrinkage cat and tenth under the shrinkage t methods. In Fig. 2d, an alternative representation lists the top 20 gene nodes under each method. The complete list is in Supplementary Table S1.

A similar pattern can be observed in the gene that ranks second, the *SFN* gene. Its marginal effect is statistically significant, as is identified by most t-statistic type methods and PINTA (Fig. 2b-d). Previous studies have reported its ability to increase cell death in breast cancer lines and found that its hypermethylation is related to silencing of the 14–3-3σ protein in epithelial breast cancer tumors [35–37]. Its property as a hub node (Fig. 2a & b) corroborates the importance of the *SFN* gene.

The next ranking gene, *ZMAT3*, also known as *Wig-1*, is identified by NetworkHub as the third-ranking gene node and by NGP-ND as the tenth (Fig. 2d). However, it is not marginally significant and not selected in the top ten by other methods (Fig. 2b). Nevertheless, the chromosome region where it is located is amplified in many tumors including breast cancer [38]. It has been reported to be a direct target of *TP53* [39], to be associated with other targets of *TP53*, such as *FAS* and 14–3-3σ protein, and to regulate the mRNA stability of *TP53* [39, 40].

Another gene worth mentioning is the well-known tumor suppressor *TP53*, the guardian of the genome [41, 42]. Since its discovery in 1979, many studies have been devoted to the investigation of its germline/somatic mutations, its sequence context, and its impact on and association with human cancers [43, 44]. Its role in the etiology of breast cancer is beyond doubt, and yet it was not identified in the top 20 under most methods (Fig. 2c and d) due to its non-differentiability in gene expression levels. The only methods that did identify it this highly were NetworkHub as fourth, Endeavour as second, PINTA as ninth and Lin’s method as ninth. It was ranked fourth by NetworkHub because it is a hub node connecting to most gene nodes in this pathway.

The top-ranked nodes for the other three pathways, mTor, Estrogen, and JAK-STAT, are *IGF1R*-*INSR*, *ESR*-node (denoted as ESR*), and *STAT*-node (STAT*), respectively. In the mTor pathway, *IGF1R*-*INSR* has similar roles as top-ranked *IGF1* does in the P53 pathway (details in supplementary Figure S2 and Table S2). The top-ranking node ESR1* in the Estrogen signaling pathway contains the *ESR1* gene (Figure S3). Research indicates that ESR1 mutations emerge during both metastatic breast cancer treatment and tumor evolution, and thus the need for a better-personalized treatment with aromatase inhibitor therapy that sequentially monitors and targets ESR1 mutations has been suggested [45]. ESR1* also ranks among the top 25% with Lin’s (second), NGP-NR (seventh), Endeavor (first), and the t-test (ninth) methods (Figure S3 and Table S3).

The top-ranking node, *STAT*-node, in the JAK-STAT pathway contains the family of STAT genes (Figure S4), which are important for mammary cell survival and tumorigenesis [46]. The expression levels of these genes are associated with breast cancer subtypes and gene *STAT1* is known to transmit information from extracellular signals to the cell nucleus [47]. Interrupted or dysregulated function of *STAT1*-*STAT3* can cause immune deficiency or development of cancer [48]. Recently, suggestions have been made for using the anti-psychotic drug pimozide to inhibit *STAT3* and *STAT5* in breast cancer patients [49, 50]. The degree of *STAT*-node is 17 and thus it is clearly a hub node. The NGP-ND and NGP-NR methods also recognize this property, ranking it among the top 25%. NGP-ND ranks it fourth and NGP-NR third (Figure S4 and Table S4), whereas with the t-test it ranks 19 out of 32 nodes and is not statistically significant.

### Epithelial ovarian carcinoma (array expression)

To demonstrate the utility of the approach using microarray expression levels, we consider here an ovarian serous cystadenocarcinoma study with data retrieved from The Cancer Genome Atlas (TCGA). After data processing and management (quality control, outlier detection, and normalization) and filtering with clinical information (tumor type, cancer stage, and ethnic group), 282 patients with complete node expression values were obtained. Among them, seventy-two did not survive over two years and were categorized in the poor prognosis group.

**Fig. 3:**
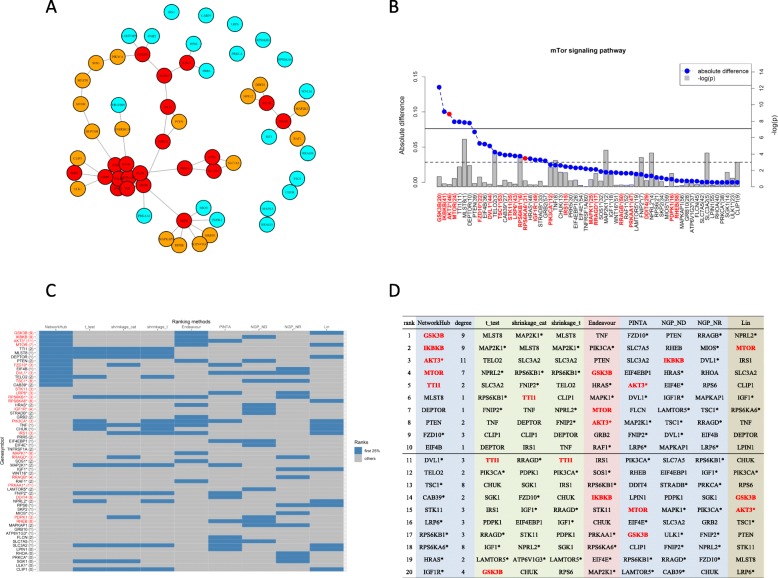
The mTor pathway for the epithelial ovarian carcinoma study. **a** Network plot. Nodes in red are hubs with degree ≥3, nodes in yellow are of degree 2, and nodes in light blue are of degree ≤1. **b** Scree plot (blue dots) of the relative influence of gene nodes (left Y-axis). The right Y-axis is the negative-logarithm-*p*-value (grey bar). The dashed red line corresponds to p = 0.05, and the solid red line to 0.05 divided by the number of nodes inside the pathway. The X-axis label indicates the gene node. A name followed by an * indicates more than one gene is contained inside this node. The number in parenthesis is the ranking determined by *p*-value. **c** Heatmap for comparing rankings under different methods. Gene nodes ranked among the first 25% are colored in grey. The nodes considered hubs are denoted in red. The number in parentheses after the gene symbol indicates the degree of the node. **d** Ranking list

The same four pathways were examined and only the mTor pathway passed the global, Fisher’s, and *t*_*all*_ gene-set association tests (Table 1). This pathway also has the highest posterior probability of association among the four, based on the Bayesian approach in Lin et al. [20]. The gene nodes in this pathway were then examined and ranked, as displayed in Fig. 3a and b. The top-ranking gene node is glycogen synthase kinase 3 beta (*GSK3B*), also a hub node, which functions in cellular processes such as proliferation and survival. This gene has been associated with drug resistance in cancer chemotherapy [51]. Higher levels of *GSK3B* are often observed in tumor tissues and overexpression of *GSK3B* can enhance tumorigenicity [52, 53]. An ongoing clinical trial (NCT03678883) is testing a GSK3B inhibitor, 9-ING-41, for treating patients with advanced cancers, including ovarian cancer.

**Table 1:** *P*-values under the first five gene-set analyses and posterior probability under the Bayesian approach for the ovarian cancer study

	Network-guided	Global	GSEA	Fisher’s	SPIA	Bayesian
JAK-STAT	0.76	0.24	0.69	0.07	0.88	0.67
P53	0.53	0.33	0.24	0.36	0.41	0.71
mTor	0.01	0.003	0.66	0.01	0.54	0.79
Estrogen	0.73	0.24	0.43	0.18	0.98	0.73

When compared with other methods, Lin’s and Endeavour also rank *GSK3B* among the top 25% (Fig. 3c); the ranks are 14 by Lin’s method and 4 by Endeavour (Fig. 3d). However, the ranks obtained by t-test, shrinkage t, shrinkage cat, PINTA, NGP-ND, and NGP-NR are not high, possibly due to the fact that *GSK3B* is not marginally significant. Similar patterns can be seen in the following three gene nodes, *IKBKB*, AKT3* and *MTOR*, which are all hub nodes and rank higher, but are not differentially expressed genes (more details are in Table S5).

### Simulation studies for ranking hub gene nodes

The following simulation studies were designed to evaluate the performance of NetworkHub in ranking the hub gene nodes. The structure of JAK-STAT pathway containing 32 nodes was considered as a prototype of the network, where two hub nodes and eight non-hub nodes were chosen as causal genes of different effect sizes. These causal nodes were then examined to see how well the procedure could prioritize them. In order to maintain the inherent biological relationship among gene nodes, sample individuals were randomly selected from the above ovarian cancer study subjects and their corresponding array expression levels were included for analysis and for generating disease status via a logit link function $\mathrm{logit}\left({p}_i\right)={\beta}_0+\sum \limits_{j=1}^{10}{\beta}_j{G}_{ij}$, where *p*_*i*_ is the disease probability of the binary disease status of this *i*-th subject. The effect sizes *β*_1_, ..., *β*_10_ under each of the four scenarios (A-D) are displayed in Table 2, including hub nodes with strong (*β*_*j*_ = 1), moderate (*β*_*j*_ = 0.5), or weak effect (*β*_*j*_ = 0.1). Under each scenario, 1000 replications were performed. In each replication, the number of subjects was 100 with 50 cases and 50 controls. Once the disease status and expression values were available, the NetworkHub procedures were carried out. Since most prioritization methods require literature mining and are not suitable for simulation studies, here we can only compare NetworkHub with three simple methods, the t-test, shrinkage cat, and shrinkage t [54]. This comparison can demonstrate the advantages when network information is included, but does not allow comparison with other network-based methods described in earlier sections.

**Table 2:** Number and effect size of the causal hub nodes and causal non-hub nodes under each scenario

Scenarios	Causal nodes			
	Hub nodes		Non-hub nodes	
	number	effect size	number	effect size
A	2	strong (*β*_*j*_ = 1)	8	weak (*β*_*j*_ = 0.1)
B	2	moderate (*β*_*j*_ = 0.5)	8	weak (*β*_*j*_ = 0.1)
C	2	weak (*β*_*j*_ = 0.1)	8	weak (*β*_*j*_ = 0.1)
D	0	null (*β*_*j*_ = 0)	0	null (*β*_*j*_ = 0)

**Fig. 4:**
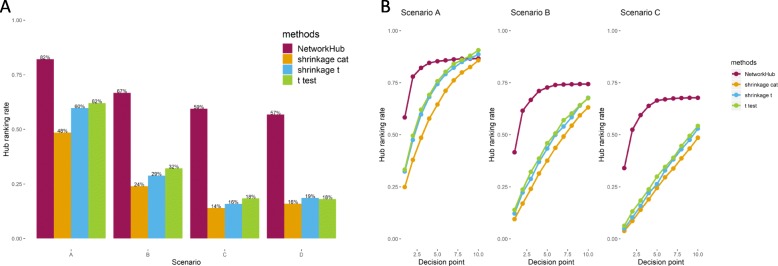
Hub ranking rates (HRR) in simulation studies. **a** The hub ranking rate of the four ranking methods under Scenarios **a**, **b**, **c**, and **d**, at decision point 3. **b** The HRR of the four ranking methods under Scenarios A-C at different decision points

Two criteria were considered for performance evaluation. The first one focuses on the ability to detect causal hub nodes, the hub ranking rate (HRR). This rate is the proportion of causal hub nodes whose rankings are less than *x* among the top *x* gene nodes: HRR = {no. of [rank(hub) ≤ *x*]} /*x*, where *x* is a predetermined decision point representing the number of influential nodes. Figure 4a shows the proportions under Scenarios A-D when *x* = 3. With the inclusion of network information, the resulting HRR is higher than the HRR obtained under other methods that do not allow inclusion of network information. This advantage is consistent across four scenarios. When no node is causal (Scenario D), the proposed method still selects the hub nodes prior to other nodes, which is expected because it is the network information that now dominates. The other three methods do not have the same tendency; their HRRs are between 16 and 19%, close to the rate expected by chance only (18.1%). Furthermore, when *x* ranges between 1 and 32, NetworkHub remains advantageous for Scenarios A-C, respectively, for strong, mild, and weak effects (Fig. 4b).

**Fig. 5:**
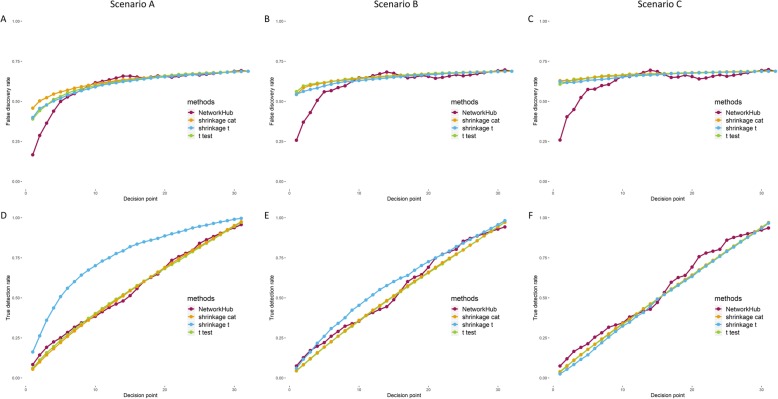
The false discovery rate (**a-c**) and true detection rate (**d-f**) of causal nodes at different decision points

Alternatively, we focus on causal nodes including both hubs and non-hubs and evaluate the false discovery rate for these causal nodes (FDR) and the true detection rate for causal nodes (TDR) among the leading *x* gene nodes. In Figs. 5a-c, note that when only the top 5 or fewer (*x* ≤ 5) genes are of interest, NetworkHub does detect the causal ones, with a lower error rate, regardless of the effect size ranging from strong (Scenario A in Fig. 5a) to mild (Scenario B in Fig. 5b), and to weak (Scenario C in Fig. 5c). For TDR, the shrinkage t-test performs the best when the effect size is strong; while others have similar rates (Scenario A in Fig. 5d). This is not surprising because we assumed only marginal effects in simulating the data. Such advantage disappears, however, when the effect size is moderate or weak (Scenarios B and C in Figs. 5e-f). In that case, all four methods performed similarly.

## Discussion

Pathways are biological systems connecting genes, proteins, and chemical substances that participate together in a molecular function. It is known that nodes inside a pathway are dependent on each other, and some nodes like hub nodes may serve as gate keepers that can maintain or disrupt this biological function. Such relationships should be considered if the aim is to rank gene nodes in the same pathway. The proposed pathway activity score integrates gene expression values, takes into account their differential status as well as their dependence, and includes available network information. In the breast and ovarian cancer applications we demonstrated that the proposed procedure NetworkHub can identify genes that have been incorporated in current standard treatments or are being evaluated in ongoing clinical trials. This procedure can provide a complementary tool when ranking a set of genes in a network structure where hub nodes are present.

Some details should be noted, however, when applying this procedure. First, when incorporating the network information, the threshold *α*_*S*_ is used to flexibly include or exclude nodes in the pathway. It has been set at the default value 1 in all the analyses. Other choices of this value change the results only slightly. In Figure S5A we calculate the HRR at different values of *α*_*S*_. Note that the HRR maintains a satisfactory level even when *α*_*S*_ is set at 0.05, remaining above 60% under Scenarios A and B. Second, the proposed ranking procedure can be applied before or after the pathway association has been tested. In Figure S5B we display the HRR based on the same simulations as in Figure S5A, but only for significantly associated pathways. The HRR becomes slightly larger, indicating little gain if a pathway association test is conducted a priori. The third issue is about the network information. The network information included in NetworkHub involves the correlation and path length *d*_*jk*_. The path length is not a measure of Euclidean distance, but rather one that is comparable to the likelihood of molecular interaction between two nodes. A smaller *d*_*jk*_ implies a larger chance of interaction.

There are issues involved in this procedure worth further investigation. First, we considered here only one dataset from a breast cancer study and one from an ovarian cancer study to demonstrate the procedure. If one aims at unraveling genetic causality for a specific disease, then the proposed NetworkHub should be applied to other datasets containing comparable diseased subjects or the integrative analysis of multi-omic data should be implemented. Second, the simulation studies conducted here only compared NetworkHub with 3 t-statistic-type tests, where sampling variation is accounted for, but no network properties are included, in the ranking procedures. This comparison did not cover other network-based methods because their execution does not include sampling variation. For instance, Endeavour does not incorporate information about gene expression, which leads to the same list of rankings in every replication across all scenarios of simulation. PINTA reads the input expression data from a dialog box online, which requires manual input and thus is not practical for simulation studies. A similar problem exists for NGP-ND and NGP-NR. Therefore, we can compare all nine methods in the two cancer studies, but not in simulation studies. Further studies may focus on modifications of those algorithms, or on other disease datasets, for broader applicability. Third, the definition of pathways in different sources may vary. The use of multiple pathway databases has been suggested because the choice of the database could impact the enrichment analysis and predictive model [55]. The need to name and annotate the pathway with a unique identification number has been called for in a comparison review of twenty-four human cell signaling pathway databases [56]. Here we adopted KEGG [19] simply for demonstration and other platforms such as String with protein-protein interaction [17] or even a user-defined pathway network can be applied straightforwardly. The codes stored in GitHub (https://github.com/Hung-Ching-Chang/NetworkHub) currently work for input from KEGG only and we are working on inclusion of other types of input. This also relates to the fourth issue, namely, when NetworkHub is to be applied in situations where a large user-defined gene-set is provided, instead of a known biological pathway. This case can arise in analysis that explores unknown relationships among gene nodes. Since the network structure is not clear in such a set, a fully connected network where all nodes are linked directly to each other may be considered. Further investigations are warranted to examine if any unnecessary edges between nodes affect the final conclusion.

## Conclusions

In summary, we proposed a network-based procedure, NetworkHub, to prioritize the gene nodes, especially the hub nodes, contained in a biological pathway network. This procedure first constructs a pathway activity score based on gene expression value, marginal effect, and network information. The network information, also termed as the intra-network association, is a function of Pearson’s correlation and minimum path length between all possible pairs of nodes, subject to a user-defined threshold for nodes to be included in this calculation. This pathway activity score was next used in a leave-one-out evaluation to prioritize the importance of the gene nodes. The application of this procedure to two cancer studies identified several important genes that have now been used in standard treatment or currently considered in clinical trials for target therapy.

## Methods

For each sample *i* (*i* = 1, ..., *N*), the pathway activity score ${T}_i^{raw}$ combines the local effect through the weight *L*_*j*_ and the topology information *S*_*j*_ derived from the *j* th gene node (*j* = 1, ..., *M*) for all *M* nodes in the same pathway as

$$ {T}_i^{raw}=\sum \limits_{j=1}^M{g}_{ij}\times \left({L}_j+{S}_j\right). $$

This score summarizes the information contained in this group of genes with three measurements: the expression level of genes *g*_*ij*_, the information *L*_*j*_ about whether the gene is differentially expressed, and the network topology information *S*_*j*_ carried by the *j* th gene in the network. The details of the last two are as follows.

### Summarizing the local effect and network information

#### Weighting the local effect

The weight of the local effect *L*_*j*_ for the *j* th gene is a weight based on its negative logarithm of *p*-values

$$ {L}_j=\frac{-\log {p}_j\times I\left({p}_j<{\alpha}_L\right)}{\sum \limits_{m=1}^M\left[-\log {p}_m\times I\left({p}_m<{\alpha}_L\right)\right]}. $$

This *p*-value results from a single-marker test such as a t-test on gene expression levels between two phenotypic groups. The indicator function *I*(*p*_*j*_ < *α*_*L*_) determines which p-values are included in the weighting system, where *α*_*L*_ is the threshold for differential expression. In other words, this local effect assigns larger weights, on an exponential scale, to genes that are statistically significant at the significance level *α*_*L*_. The default is *α*_*L*_ = 0.05. When *α*_*L*_ = 1, the summation of all *L*_*j*_ becomes the scaled test statistic of Fisher’s method for gene set analysis [7, 57]. Note that the denominator is included so that the local effect weights sum to 1.

#### Weighting network information

The topology information measure *S*_*j*_ at the *j* th node is defined as

$$ {S}_j=\frac{\sum \limits_{k=1,k\ne j}^M\frac{\mid {\rho}_{jk}\mid }{d_{jk}}\times I\left({d}_{jk}<D,{p}_j<{\alpha}_S\right)}{\sum \limits_{m=1}^M\left\{\sum \limits_{k=1,k\ne m}^M\frac{\mid {\rho}_{mk}\mid }{d_{mk}}\times I\left({d}_{mk}<D,{p}_m<{\alpha}_S\right)\right\}}. $$

This measure contains three elements: (1) the pairwise absolute Pearson’s correlation ∣*ρ*_*jk*_∣ between the current node *j* and the rest of the nodes, (2) the path length *d*_*jk*_ for the closeness between nodes *j* and *k*, and (3) the threshold *D* which controls the size of the topology influence. The closeness *d*_*jk*_ here is defined as the minimum steps/edges between two nodes in the network. It is used to balance the effect of correlation. For example, when two nodes are highly correlated but distant, it implies they are less likely to interact directly, and therefore the correlation is down-weighted by the path length.

The third component, the threshold *D*, regulates the type and size of the topological influence. It can be a very large number so that all gene nodes contribute to this measure. Alternatively, it can be determined to include only first-degree neighbors such as the immediately adjacent up- and down-stream genes, or genes whose coding protein is being directly regulated.

The number *α*_*S*_ in *S*_*j*_ also serves as a gatekeeper to control the number of genes involved in the topological contribution. When the default value *α*_*S*_ = 1 is used, all other nodes, whether differentially expressed or not, will contribute to the topological influence of this gene. If one chooses a small value for *α*_*S*_, say 0.05, then only differentially expressed genes are included. Again, the denominator in *S*_*j*_ guarantees these weights sum to 1.

In brief, this measure *S*_*j*_ has several features. First, it incorporates the relationship among genes through the use of correlation, which relaxes the traditional assumption of independence. Second, this correlation is balanced with the inclusion of the length *d*_*jk*_. In other words, a large correlation between two genes far apart in the network will be moderated by a correspondingly large *d*_*jk*_; while the correlation between two genes in a direct regulation will not. Third, the threshold *D* for the minimum distance offers the flexibility to include either all genes or only the nearby ones in the evaluation of network influence. Finally, the number *α*_*S*_ provides the chance to include only genes passing the single-marker association test.

### Regularization of effect direction

When combining the gene expressions in a network, caution should be taken if there exist genes negatively correlated with each other. A direct summation of the expression levels without taking into account their interrelation may underestimate the strength of the effect. Therefore, for gene *j*, if its t-test statistic is negative (*t*_*j*_ < 0) in the single-marker test, then the expression values will be regularized by its maximum value *O*_*j*_, where *O*_*j*_ = max {*g*_1*j*_, *g*_2*j*_, ..., *g*_*Nj*_} is the maximum across all observed gene expressions from *N* samples at the same gene *j*, and the expression to be used for calculating the network score becomes

$$ {g}_{ij}^R=\Big\{{\displaystyle \begin{array}{c}{g}_{ij},\kern3.75em \mathrm{if}\ {t}_j\ge 0\\ {}{O}_j-{g}_{ij}+\varepsilon, \kern0.5em \mathrm{if}\ {t}_j<0\end{array}}. $$

Here the *ε* is a small positive number, say 0.001, to guard against a value of zero. This standardization leads to our proposed pathway activity score (PAS) for the network

$$ {T}_i=\sum \limits_{j=1}^M{g}_{ij}^R\times \left({L}_j+{S}_j\right). $$

This regularization avoids excessive cancellation in summing ${g}_{i{j}_1}$ and ${g}_{i{j}_2}$ when they are negatively correlated and, instead, gives a relatively large value of PAS to reflect the higher degree of activity level in this pathway for sample *i*. Here the t statistic *t*_*j*_ is used as a reference for regularization; other alternatives include the difference in the average expression level between two phenotypic groups or the fold-change between the two groups.

### Gene-ranking with leave-one-out evaluation

To rank the gene nodes, we first compute the PAS with the procedures described above for each sample, and then calculate the difference in average PAS between two phenotypic groups (grp1 and grp2), *t*_*all*_, standardized by standard errors, *se*, corresponding to each group,

$$ {t}_{all}=\frac{{\overline{T}}_{grp1}-{\overline{T}}_{grp2}}{\sqrt{{\left[ se\left({T}_{i,i\in grp1}\right)\right]}^2+{\left[ se\left({T}_{i,i\in grp2}\right)\right]}^2}}. $$

Next, we rearrange the network by leaving one gene node out, evaluate again the local weight and topology measure for each node in the new network with the *M* − 1 nodes, derive the corresponding PAS for each sample, denoted as *T*_*i*(−*j*)_, and then calculate the standardized difference in average PAS as *t*_(−*j*)_, where *j* is the index of the node removed.

Once all nodes are visited in turn, and the corresponding standardized differences have been computed, their magnitudes are then compared with the original *t*_*all*_, by taking the absolute difference *Q*_*j*_ =  ∣ *t*_*all*_ − *t*_(−*j*)_∣. These values can be ordered and displayed in a scree plot to facilitate analysis. A large value indicates a substantial change when the gene node is removed from the pathway network, while a small value implies little perturbation with this deletion. The scree plot of the sorted absolute differences *Q*_(*j*)_—i.e., the order statistics—from largest to smallest, can provide the ranking in terms of importance, indicating which nodes may be useful in target therapy or drug development within this set of interest. This is the NetworkHub procedure. In addition, if a cut-off is needed to select influential gene nodes, the inflection point on the curve, defined as the first ordered *Q*_(*j*)_ where (*Q*_(*j* + 1)_ − *Q*_(*j*)_) − (*Q*_(*j*)_ − *Q*_(*j* − 1)_) becomes negative, can be used where all nodes before *Q*_(*j*)_ are considered as influential. The R code for performing the pathway test and gene-ranking, an example, and the document files are freely available online (https://github.com/Hung-Ching-Chang/NetworkHub).

## Supplementary information

**Additional file 1: Tables S1-S5**. for detailed lists of rankings in each pathway under different methods.

**Additional file 2: Figure S1.** Boxplots of local weights (left column) and topology weights (right column). Here the thresholds are set at conservative values: 0.05 for *α*_*L*_, 1 for *α*_*S*_, and no limit for *D* so that all nodes are included. **Figure S2:** Analyses of the mTor pathway for the breast cancer study. **Figure S3:** Analyses of the estrogen pathway for the breast cancer study. **Figure S4:** Analyses of the JAK-STAT pathway for the breast cancer study. **Figure S5:** The hub ranking rate of NetworkHub corresponding to different threshold values before and after the pathway association test, under four scenarios. **A:** Before. **B:** After.

## Data Availability

The breast cancer data are publicly available and can be downloaded from NCBI GEO database (GSE69240). The ovarian carcinoma array expression levels are also freely downloadable from The Cancer Genome Atlas (TCGA). The R code for implementation and tutorial documents are freely available and can be downloaded from https://github.com/Hung-Ching-Chang/NetworkHub.

## Abbreviations

FDR: False discovery rate
GSEA: Gene set enrichment analysis
HRR: Hub ranking rate
KEGG: Kyoto encyclopedia of genes and genomes
ND: Networked differential expression
NetworkHub: Network hub prioritization method
NGP: Network gene prioritizer
NR: Network rewiring
TCGA: The cancer genome atlas
TDR: True detection rate for causal nodes
