# Supplementary material for: Network hub-node prioritization of gene regulation with intra-network association
Source: BMC Bioinformatics. 2020 Mar 12;21:101. doi: 10.1186/s12859-020-3444-7 (PMC7069025; doi:10.1186/s12859-020-3444-7)
Supplement: Supplementary file 1 — Additional file 1: Tables S1-S5. for detailed lists of rankings in each pathway under different methods. [file 12859_2020_3444_MOESM1_ESM.docx]

**Table S1:** Ranking of gene nodes in P53 pathway by different methods for the breast cancer study.

|  | NetworkHub | degree | t_test | shrinkage_cat | shrinkage_t | Endeavour | PINTA | NGP_ND | NGP_NR | Lin |
| --- | --- | --- | --- | --- | --- | --- | --- | --- | --- | --- |
| 1 | **IGF1** | 1 | CDK1 | **SFN** | CDK1 | CDKN1A | BBC3 | BAI1 | MDM4 | GTSE1 |
| 2 | **SFN** | 3 | RRM2B* | **IGF1** | **SFN** | **TP53** | PMAIP1 | RRM2B* | SIAH1 | CHEK1 |
| 3 | **ZMAT3** | 1 | CDKN1A | CDK1 | TNFRSF10B | ATM | **SFN** | CASP9 | **IGF1** | CCNB1* |
| 4 | **TP53** | 38 | PMAIP1 | BBC3 | RRM2B* | **MDM2** | SERPINB5 | TSC2 | THBS1 | BBC3 |
| 5 | **MDM2** | 3 | GTSE1 | RRM2B* | CDKN1A | CHEK2 | CDKN1A | CCNE1* | CCNB1* | CDK1 |
| 6 | FAS | 1 | TP73 | SERPINE1 | SERPINE1 | PTEN | SERPINE1 | BID | CHEK1 | BAX |
| 7 | GADD45G* | 3 | TNFRSF10B | TNFRSF10B | BBC3 | CDK4* | TNFRSF10B | CASP3 | CCNE1* | CCND1* |
| 8 | RRM2B* | 1 | CCNB1* | PMAIP1 | GADD45G* | CHEK1 | GADD45G* | CDK1 | CHEK2 | CASP8 |
| 9 | GTSE1 | 1 | **SFN** | GTSE1 | PMAIP1 | CDKN2A | **TP53** | IGFBP3 | GTSE1 | SHISA5 |
| 10 | CDKN2A | 1 | FAS | DDB2 | **IGF1** | CCND1* | CD82 | **ZMAT3** | SERPINE1 | **IGF1** |
| 11 | IGFBP3 | 2 | SERPINE1 | CDKN1A | GTSE1 | CDK2 | BID | RCHY1 | CDKN1A | **TP53** |
| 12 | CDK1 | 3 | SERPINB5 | CDKN2A | FAS | CASP9 | IGFBP3 | FAS | SESN3* | STEAP3 |
| 13 | BAI1 | 1 | GADD45G* | TP73 | CCNB1* | MDM4 | CYCS | CCNB1* | CDK2 | TNFRSF10B |
| 14 | CD82 | 1 | CCNE1* | GADD45G* | TP73 | APAF1 | **ZMAT3** | CCND1* | PPM1D | ATM |
| 15 | PERP | 1 | IGF1 | SERPINB5 | CHEK2 | TSC2 | THBS1 | CHEK1 | BBC3 | APAF1 |
| 16 | CCNG1* | 1 | BAX | SIAH1 | BAX | TP73 | RCHY1 | CDK4* | PIDD | **SFN** |
| 17 | CYCS | 4 | BBC3 | FAS | SERPINB5 | ATR | CDK2 | GTSE1 | CDK1 | CD82 |
| 18 | DDB2 | 1 | CASP9 | SESN3* | CCNE1* | GTSE1 | PERP | CDKN2A | CASP8 | PMAIP1 |
| 19 | ATM | 2 | CHEK1 | CASP9 | CASP9 | CASP8 | CASP3 | TNFRSF10B | SHISA5 | SERPINE1 |
| 20 | SIAH1 | 2 | CHEK2 | CASP8 | CASP8 | PPM1D | CCNG1* | BBC3 | PTEN | CDKN1A |
| 21 | TSC2 | 1 | CASP8 | CCNG1* | CHEK1 | CCNB1* | PPM1D | PPM1D | TP53AIP1 | CHEK2 |
| 22 | SHISA5 | 1 | CD82 | CDK4* | CDKN2A | CCNE1* | CCNE1* | SERPINE1 | **MDM2** | CYCS |
| 23 | CDKN1A | 5 | CDKN2A | BAX | THBS1 | RFWD2 | CDK4* | RFWD2 | **SFN** | TP73 |
| 24 | CDK2 | 2 | DDB2 | THBS1 | CD82 | RCHY1 | EI24* | DDB2 | **TP53** | CDK2 |
| 25 | STEAP3 | 1 | RCHY1 | CCNB1* | BID | FAS | MDM4 | CCNG1* | CDKN2A | RRM2B* |
| 26 | PTEN | 1 | TP53AIP1 | ATR | CYCS | CD82 | PTEN | CASP8 | TNFRSF10B | CASP9 |
| 27 | CCND1* | 2 | CYCS | MDM4 | DDB2 | THBS1 | ATR | PERP | CD82 | BID |
| 28 | CCNB1* | 3 | BID | **MDM2** | IGFBP3 | TP53AIP1 | RFWD2 | STEAP3 | CDK4* | CDK4* |
| 29 | ATR | 2 | SHISA5 | CCND1* | CDK4* | BAX | GORAB | BAX | EI24* | SIAH1 |
| 30 | SESN3* | 1 | THBS1 | SHISA5 | SHISA5 | CDK1 | ATM | SHISA5 | DDB2 | CDKN2A |
| 31 | THBS1 | 1 | CDK4* | **TP53** | TP53AIP1 | SIAH1 | TSC2 | PTEN | CASP9 | PERP |
| 32 | MDM4 | 2 | SESN3* | TP53AIP1 | RCHY1 | TNFRSF10B | SIAH1 | CHEK2 | FAS | GADD45G* |
| 33 | TP73 | 1 | PIDD | BAI1 | **ZMAT3** | BBC3 | SESN3* | TP73 | CCNG1* | FAS |
| 34 | SERPINB5 | 1 | ATM | CHEK1 | ATM | GADD45G* | RRM2B* | PIDD | **ZMAT3** | SERPINB5 |
| 35 | TNFRSF10B | 2 | IGFBP3 | CCNE1* | SESN3* | EI24* | SHISA5 | CYCS | GORAB | RCHY1 |
| 36 | TP53AIP1 | 2 | PERP | PIDD | PERP | **IGF1** | DDB2 | CDK2 | TSC2 | TP53AIP1 |
| 37 | PMAIP1 | 1 | APAF1 | RCHY1 | APAF1 | CASP3 | CCND1* | PMAIP1 | CCND1* | DDB2 |
| 38 | BID | 1 | SIAH1 | CD82 | CDK2 | RRM2B* | CASP9 | GADD45G* | SERPINB5 | IGFBP3 |
| 39 | CHEK2 | 2 | **ZMAT3** | BID | SIAH1 | PIDD | BAI1 | ATR | BAX | GORAB |
| 40 | CDK4* | 2 | CDK2 | CYCS | PIDD | DDB2 | CHEK1 | SERPINB5 | IGFBP3 | THBS1 |
| 41 | PPM1D | 1 | **TP53** | GORAB | **TP53** | PMAIP1 | CCNB1* | ATM | ATR | **ZMAT3** |
| 42 | CCNE1* | 2 | STEAP3 | RFWD2 | CCNG1* | **ZMAT3** | **IGF1** | CD82 | STEAP3 | ATR |
| 43 | CASP9 | 3 | PTEN | PERP | STEAP3 | CCNG1* | CDKN2A | **SFN** | BAI1 | SESN3* |
| 44 | BAX | 1 | CCNG1* | CDK2 | PTEN | CYCS | TP73 | SESN3* | PMAIP1 | CCNG1* |
| 45 | CHEK1 | 2 | TSC2 | STEAP3 | TSC2 | BAI1 | CDK1 | EI24* | RRM2B* | EI24* |
| 46 | CASP8 | 2 | BAI1 | PPM1D | BAI1 | SHISA5 | TP53AIP1 | APAF1 | ATM | RFWD2 |
| 47 | SERPINE1 | 1 | CASP3 | PTEN | CCND1* | SERPINE1 | APAF1 | SIAH1 | BID | PIDD |
| 48 | EI24* | 1 | **MDM2** | CHEK2 | CASP3 | SESN3* | BAX | CDKN1A | RCHY1 | MDM4 |
| 49 | RFWD2 | 1 | RFWD2 | EI24* | **MDM2** | PERP | CASP8 | **MDM2** | CASP3 | TSC2 |
| 50 | APAF1 | 1 | ATR | ATM | EI24* | **SFN** | CHEK2 | **TP53** | RFWD2 | PTEN |
| 51 | RCHY1 | 1 | EI24* | APAF1 | ATR | BID | FAS | MDM4 | PERP | PPM1D |
| 52 | CASP3 | 2 | CCND1* | CASP3 | GORAB | IGFBP3 | GTSE1 | **IGF1** | TP73 | CASP3 |
| 53 | BBC3 | 2 | GORAB | IGFBP3 | RFWD2 | STEAP3 | **MDM2** | THBS1 | APAF1 | BAI1 |
| 54 | PIDD | 1 | PPM1D | **ZMAT3** | PPM1D | SERPINB5 | PIDD | TP53AIP1 | GADD45G* | CCNE1* |
| 55 | GORAB | 0 | MDM4 | TSC2 | MDM4 | GORAB | STEAP3 | GORAB | CYCS | **MDM2** |

**Table S2:** Ranking of gene nodes in mTor pathway by different methods for the breast cancer study.

|  | NetworkHub | degree | t_test | shrinkage_cat | shrinkage_t | Endeavour | PINTA | NGP_ND | NGP_NR | Lin |
| --- | --- | --- | --- | --- | --- | --- | --- | --- | --- | --- |
| 1 | **IGF1R*** | 4 | RPS6KA6* | SESN2 | TNF | PIK3CA* | LAMTOR5* | MIOS* | DEPTOR | **IGF1** |
| 2 | **IKBKB** | 3 | SLC7A5 | TNF | SESN2 | PTEN | FZD10* | STK11 | RPS6KB1* | MTOR |
| 3 | **TSC1*** | 9 | CLIP1 | RPS6KA6* | SLC3A2 | TNF | SESN2 | RPS6KB1* | FZD10* | EIF4EBP1 |
| 4 | **TELO2** | 2 | MIOS* | SKP2 | SKP2 | GSK3B | SLC7A5 | PIK3CA* | PRR5 | FLCN |
| 5 | **IGF1** | 1 | FLCN | **IGF1** | RPS6KA6* | HRAS* | RRAGD* | MTOR | FLCN | DEPTOR |
| 6 | STRADB* | 2 | SKP2 | SLC3A2 | SLC7A5 | MAPK1* | EIF4EBP1 | **TELO2** | MAPKAP1 | SLC3A2 |
| 7 | MAPK1* | 4 | SESN2 | DVL1* | MAP2K1* | PDPK1 | SLC3A2 | SOS1* | PIK3CA* | WNT4* |
| 8 | CAB39* | 2 | TNF | SLC7A5 | STRADB* | MTOR | AKT3* | DDIT4 | RNF152 | STRADB* |
| 9 | RRAGB* | 5 | STRADB* | FLCN | FLCN | GRB2 | DVL1* | CAB39* | EIF4EBP1 | SKP2 |
| 10 | RICTOR | 3 | EIF4EBP1 | SGK1 | RHEB | AKT3* | RNF152 | RNF152 | EIF4E* | TNF |
| 11 | TNF | 1 | RHEB | MIOS* | **IGF1** | IRS1 | FLCN | RPS6KA6* | **IGF1R*** | CLIP1 |
| 12 | IRS1 | 3 | **IGF1** | RHEB | CLIP1 | **IKBKB** | MAP2K1* | EIF4EBP1 | ULK1* | RAF1* |
| 13 | DVL1* | 3 | DVL1* | PRKCA* | SGK1 | RPS6KB1* | RHEB | PDPK1 | RRAGB* | SLC7A5 |
| 14 | RRAGD* | 3 | SGK1 | DEPTOR | EIF4B | RAF1* | FNIP2* | PRR5 | RHOA | TBC1D7 |
| 15 | PTEN | 2 | MAP2K1* | WNT4* | EIF4E* | MIOS* | PIK3CA* | SGK1 | HRAS* | RHEB |
| 16 | MAP2K1* | 2 | EIF4E* | RPS6 | EIF4EBP1 | SOS1* | LRP6* | NPRL2* | PTEN | EIF4B |
| 17 | MTOR | 7 | RAF1* | CHUK | MIOS* | STK11 | DDIT4 | FLCN | LAMTOR5* | RPS6 |
| 18 | RHEB | 2 | RRAGD* | HRAS* | RRAGD* | PRKAA1* | MTOR | PTEN | MAPK1* | FZD10* |
| 19 | AKT3* | 5 | SLC3A2 | DDIT4 | RHOA | AKT1S1 | RICTOR | LAMTOR5* | CHUK | RPS6KB1* |
| 20 | GSK3B | 3 | LRP6* | EIF4EBP1 | LPIN1 | CHUK | LPIN1 | RHEB | AKT3* | SESN2 |
| 21 | MLST8 | 1 | TNFRSF1A | **IKBKB** | RAF1* | MAPKAP1 | EIF4E* | MAP2K1* | RICTOR | PRR5 |
| 22 | PDPK1 | 3 | LPIN1 | MTOR | DVL1* | RICTOR | GSK3B | **TSC1*** | **TSC1*** | MAP2K1* |
| 23 | TBC1D7 | 9 | EIF4B | MAP2K1* | PRR5 | SKP2 | RPS6KB1* | STRADB* | RAF1* | SGK1 |
| 24 | PRR5 | 1 | PRKCA* | RICTOR | TNFRSF1A | FLCN | ULK1* | RICTOR | LRP6* | DDIT4 |
| 25 | DDIT4 | 2 | MAPK1* | LAMTOR5* | DDIT4 | PRKCA* | RPTOR | MLST8 | STK11 | RICTOR |
| 26 | RAF1* | 2 | GRB10 | **IGF1R*** | DEPTOR | MAP2K1* | CLIP1 | FZD10* | PDPK1 | RRAGB* |
| 27 | DEPTOR | 1 | FNIP2* | RRAGD* | RICTOR | EIF4E* | PRKAA1* | LRP6* | SESN2 | LPIN1 |
| 28 | EIF4B | 1 | RHOA | EIF4B | LRP6* | GRB10 | PRKCA* | RRAGD* | SGK1 | AKT3* |
| 29 | PIK3CA* | 3 | SOS1* | STRADB* | AKT3* | TNFRSF1A | RAF1* | CLIP1 | ATP6V1C2* | RHOA |
| 30 | RNF152 | 1 | HRAS* | EIF4E* | RPS6 | **IGF1R*** | **TSC1*** | LPIN1 | **IKBKB** | EIF4E* |
| 31 | CHUK | 1 | GSK3B | CAB39* | PRKCA* | RPS6KA6* | CAB39* | DVL1* | EIF4B | PIK3CA* |
| 32 | NPRL2* | 2 | DDIT4 | AKT3* | GRB10 | LRP6* | RHOA | AKT1S1 | DVL1* | LAMTOR5* |
| 33 | LAMTOR5* | 3 | PRR5 | PTEN | HRAS* | MLST8 | CHUK | GSK3B | FNIP2* | LRP6* |
| 34 | GRB2 | 2 | CHUK | FZD10* | FNIP2* | CAB39* | TNFRSF1A | CHUK | CAB39* | GRB10 |
| 35 | FNIP2* | 2 | RICTOR | LPIN1 | SOS1* | CLIP1 | RPS6 | EIF4E* | DDIT4 | GSK3B |
| 36 | RPTOR | 2 | RPS6 | RAF1* | CHUK | **TSC1*** | MAPK1* | ATP6V1C2* | **IGF1** | CHUK |
| 37 | SESN2 | 1 | AKT3* | PRR5 | MAPK1* | PRR5 | AKT1S1 | MAPK1* | WNT4* | RNF152 |
| 38 | MIOS* | 2 | DEPTOR | GRB10 | GSK3B | STRADB* | EIF4B | SLC3A2 | STRADB* | SLC38A9 |
| 39 | RPS6KB1* | 3 | MLST8 | SOS1* | MLST8 | RHOA | **IGF1R*** | WNT4* | SOS1* | FNIP2* |
| 40 | SLC38A9 | 1 | LAMTOR5* | CLIP1 | RNF152 | DEPTOR | **IKBKB** | TTI1 | GRB2 | TNFRSF1A |
| 41 | WNT4* | 2 | RNF152 | GRB2 | LAMTOR5* | RPTOR | RRAGB* | TBC1D7 | GSK3B | DVL1* |
| 42 | SOS1* | 2 | FZD10* | LRP6* | FZD10* | **IGF1** | STK11 | RPTOR | TNFRSF1A | CAB39* |
| 43 | MAPKAP1 | 1 | **TELO2** | NPRL2* | SLC38A9 | ULK1* | SOS1* | GRB2 | SLC7A5 | RRAGD* |
| 44 | TNFRSF1A | 2 | **IKBKB** | IRS1 | ATP6V1C2* | FZD10* | TTI1 | GRB10 | **TELO2** | MAPK1* |
| 45 | RPS6KA6* | 3 | **IGF1R*** | GSK3B | MTOR | TBC1D7 | PDPK1 | RHOA | IRS1 | MLST8 |
| 46 | FZD10* | 3 | SLC38A9 | FNIP2* | TBC1D7 | RNF152 | NPRL2* | ULK1* | MIOS* | PRKCA* |
| 47 | GRB10 | 1 | CAB39* | PRKAA1* | **TELO2** | RHEB | PTEN | RPS6 | NPRL2* | **TELO2** |
| 48 | TTI1 | 2 | ATP6V1C2* | STK11 | **IKBKB** | TTI1 | MIOS* | EIF4B | CLIP1 | SOS1* |
| 49 | FLCN | 2 | TBC1D7 | TBC1D7 | WNT4* | RPS6 | HRAS* | SESN2 | TNF | MAPKAP1 |
| 50 | AKT1S1 | 3 | WNT4* | **TSC1*** | CAB39* | SESN2 | ATP6V1C2* | **IKBKB** | AKT1S1 | RPTOR |
| 51 | SKP2 | 1 | MTOR | RPTOR | **IGF1R*** | SLC3A2 | GRB2 | AKT3* | MTOR | PRKAA1* |
| 52 | ATP6V1C2* | 1 | PTEN | MAPK1* | PTEN | WNT4* | IRS1 | FNIP2* | LPIN1 | PDPK1 |
| 53 | STK11 | 3 | ULK1* | ULK1* | ULK1* | **TELO2** | RPS6KA6* | MAPKAP1 | TBC1D7 | MIOS* |
| 54 | PRKAA1* | 5 | NPRL2* | AKT1S1 | MAPKAP1 | NPRL2* | TBC1D7 | SLC7A5 | SKP2 | **IGF1R*** |
| 55 | LRP6* | 3 | IRS1 | PIK3CA* | IRS1 | EIF4EBP1 | SLC38A9 | **IGF1R*** | SLC38A9 | STK11 |
| 56 | EIF4EBP1 | 1 | MAPKAP1 | PDPK1 | NPRL2* | RRAGB* | **TELO2** | TNF | RPS6KA6* | TTI1 |
| 57 | EIF4E* | 1 | STK11 | TNFRSF1A | RPTOR | LPIN1 | DEPTOR | PRKAA1* | GRB10 | IRS1 |
| 58 | RPS6 | 1 | GRB2 | MLST8 | AKT1S1 | EIF4B | WNT4* | SKP2 | TTI1 | HRAS* |
| 59 | SLC7A5 | 1 | RPS6KB1* | RNF152 | STK11 | SLC38A9 | STRADB* | RAF1* | MAP2K1* | ULK1* |
| 60 | SLC3A2 | 1 | PIK3CA* | TTI1 | PIK3CA* | RRAGD* | PRR5 | SLC38A9 | SLC3A2 | GRB2 |
| 61 | HRAS* | 2 | RPTOR | SLC38A9 | GRB2 | ATP6V1C2* | SKP2 | DEPTOR | RHEB | **IKBKB** |
| 62 | LPIN1 | 0 | AKT1S1 | ATP6V1C2* | RPS6KB1* | FNIP2* | **IGF1** | IRS1 | PRKAA1* | RPS6KA6* |
| 63 | RHOA | 0 | RRAGB* | **TELO2** | TTI1 | SLC7A5 | GRB10 | **IGF1** | MLST8 | NPRL2* |
| 64 | PRKCA* | 0 | TTI1 | RPS6KB1* | RRAGB* | DVL1* | MAPKAP1 | TNFRSF1A | RRAGD* | ATP6V1C2* |
| 65 | SGK1 | 0 | PRKAA1* | RHOA | PDPK1 | DDIT4 | MLST8 | PRKCA* | PRKCA* | **TSC1*** |
| 66 | ULK1* | 0 | PDPK1 | MAPKAP1 | PRKAA1* | SGK1 | SGK1 | HRAS* | RPS6 | AKT1S1 |
| 67 | CLIP1 | 0 | **TSC1*** | RRAGB* | **TSC1*** | LAMTOR5* | TNF | RRAGB* | RPTOR | PTEN |

**Table S3:** Ranking of gene nodes in Estrogen pathway by different methods for the breast cancer study.

|  | NetworkHub | degree | t_test | shrinkage_cat | shrinkage_t | Endeavour | PINTA | NGP_ND | NGP_NR | Lin |
| --- | --- | --- | --- | --- | --- | --- | --- | --- | --- | --- |
| 1 | **ESR1*** | 13 | EGFR | HBEGF | HBEGF | **ESR1*** | HBEGF | **BCL2** | GNAS | **BCL2** |
| 2 | **TFF1** | 2 | TGFA | PLCB1* | TGFA | PIK3CA* | KRT23* | RARA | HBEGF | **ESR1*** |
| 3 | **BCL2** | 2 | HBEGF | HSPA1A* | HSPA1A* | EGFR | SHC2* | FOS* | MMP2* | **PGR** |
| 4 | **ADCY1*** | 3 | GPER1 | TGFA | MAP2K1* | HRAS* | AKT3* | KCNJ3* | **ADCY1*** | TGFA |
| 5 | **PGR** | 2 | RAF1 | **ADCY1*** | EGFR | GRB2 | MAP2K1* | KRT23* | **ESR1*** | GPER1 |
| 6 | PLCB1* | 3 | MAP2K1* | MAP2K1* | RAF1 | MAPK1* | NOS3 | **ESR1*** | PLCB1* | ITPR1* |
| 7 | HBEGF | 2 | PRKACA* | EGFR | **ADCY1*** | TGFA | PIK3CA* | GNAS | **BCL2** | AKT3* |
| 8 | NOS3 | 3 | PLCB1* | PRKACA* | KRT23* | RAF1 | SRC | EBAG9 | CALML6* | **ADCY1*** |
| 9 | PRKCD | 2 | **ESR1*** | GPER1 | GPER1 | AKT3* | RAF1 | CREB3* | KCNJ3* | KRT23* |
| 10 | RAF1 | 3 | **ADCY1*** | SHC2* | HSP90AA1* | SOS1* | CREB3* | HSPA1A* | GNAI1* | RARA |
| 11 | CALML6* | 1 | KRT23* | CALML6* | **TFF1** | SRC | FOS* | SRC | NOS3 | **TFF1** |
| 12 | SRC | 5 | **TFF1** | RAF1 | PLCB1* | **TFF1** | MAPK1* | **ADCY1*** | AKT3* | HBEGF |
| 13 | CTSD | 2 | KCNJ3* | EBAG9 | PRKACA* | PRKACA* | HSP90AA1* | MAPK1* | FOS* | EGFR |
| 14 | MMP2* | 2 | MAPK1* | AKT3* | **ESR1*** | NCOA2* | GNAQ | GNAQ | GPER1 | RAF1 |
| 15 | RARA | 2 | **PGR** | KRT23* | FOS* | MAP2K1* | SOS1* | TGFA | RARA | MAP2K1* |
| 16 | PRKACA* | 4 | CALML6* | CTSD | AKT3* | KCNJ3* | NCOA2* | **PGR** | SOS1* | HSP90AA1* |
| 17 | GNAS | 3 | SOS1* | MMP2* | KCNJ3* | **PGR** | FKBP4* | FKBP4* | PIK3CA* | CREB3* |
| 18 | AKT3* | 2 | HSP90AA1* | ITPR1* | **PGR** | HSP90AA1* | MMP2* | AKT3* | **PGR** | GNAI1* |
| 19 | FOS* | 1 | HRAS* | HRAS* | CALML6* | HBEGF | PRKACA* | PRKCD | RAF1 | KCNJ3* |
| 20 | HRAS* | 3 | HSPA1A* | KCNJ3* | HRAS* | **BCL2** | HRAS* | CALML6* | SHC2* | PIK3CA* |
| 21 | KRT23* | 2 | AKT3* | **PGR** | SOS1* | PRKCD | GRB2 | GPER1 | ITPR1* | PRKCD |
| 22 | SHC2* | 2 | SRC | FOS* | MAPK1* | ITPR1* | GNAS | SOS1* | MAP2K1* | HSPA1A* |
| 23 | EGFR | 3 | SHC2* | **TFF1** | SRC | NOS3 | EBAG9 | HBEGF | CREB3* | SRC |
| 24 | EBAG9 | 2 | PRKCD | PRKCD | RARA | GNAS | CTSD | GABBR1 | PRKCD | GNAS |
| 25 | ITPR1* | 1 | FOS* | GRB2 | PRKCD | CALML6* | RARA | PLCB1* | HSPA1A* | EBAG9 |
| 26 | CREB3* | 3 | **BCL2** | **ESR1*** | NOS3 | PLCB1* | PRKCD | EGFR | GRB2 | NOS3 |
| 27 | SOS1* | 2 | RARA | HSP90AA1* | SHC2* | GNAQ | **BCL2** | HSP90AA1* | SRC | MAPK1* |
| 28 | KCNJ3* | 2 | NCOA2* | SRC | CTSD | EBAG9 | ITPR1* | NCOA2* | GNAQ | PLCB1* |
| 29 | MAP2K1* | 2 | GNAS | RARA | **BCL2** | GNAI1* | **PGR** | SHC2* | **TFF1** | CTSD |
| 30 | TGFA | 2 | FKBP4* | NOS3 | NCOA2* | GABBR1 | **ESR1*** | MAP2K1* | HSP90AA1* | FOS* |
| 31 | GRB2 | 2 | GNAQ | CREB3* | FKBP4* | CREB3* | **ADCY1*** | RAF1 | KRT23* | CALML6* |
| 32 | GPER1 | 2 | NOS3 | GNAS | GABBR1 | KRT23* | **TFF1** | ITPR1* | MAPK1* | SOS1* |
| 33 | GNAI1* | 2 | EBAG9 | MAPK1* | GNAS | RARA | GPER1 | **TFF1** | TGFA | SHC2* |
| 34 | GABBR1 | 1 | CTSD | GABBR1 | EBAG9 | **ADCY1*** | CALML6* | GRB2 | GABBR1 | GABBR1 |
| 35 | NCOA2* | 9 | GABBR1 | SOS1* | GNAQ | FOS* | KCNJ3* | CTSD | EGFR | MMP2* |
| 36 | PIK3CA* | 3 | ITPR1* | GNAI1* | MMP2* | CTSD | PLCB1* | GNAI1* | NCOA2* | GNAQ |
| 37 | MAPK1* | 3 | GNAI1* | PIK3CA* | ITPR1* | FKBP4* | EGFR | HRAS* | PRKACA* | GRB2 |
| 38 | GNAQ | 1 | MMP2* | FKBP4* | GNAI1* | MMP2* | TGFA | MMP2* | CTSD | NCOA2* |
| 39 | HSP90AA1* | 0 | GRB2 | **BCL2** | GRB2 | SHC2* | GNAI1* | PRKACA* | FKBP4* | HRAS* |
| 40 | FKBP4* | 0 | PIK3CA* | NCOA2* | PIK3CA* | HSPA1A* | GABBR1 | NOS3 | HRAS* | PRKACA* |
| 41 | HSPA1A* | 0 | CREB3* | GNAQ | CREB3* | GPER1 | HSPA1A* | PIK3CA* | EBAG9 | FKBP4* |

**Table S4:** Ranking of gene nodes in JAK-STAT pathway by different methods for the breast cancer study.

|  | NetworkHub | degree | t_test | shrinkage_cat | shrinkage_t | Endeavour | PINTA | NGP_ND | NGP_NR | Lin |
| --- | --- | --- | --- | --- | --- | --- | --- | --- | --- | --- |
| 1 | **STAT1*** | 17 | CDKN1A | IL24* | IL24* | PIK3CA* | CNTF* | PTPN2 | AOX1 | MTOR |
| 2 | **FHL1** | 1 | PTPN11 | CDKN1A | CDKN1A | CDKN1A | IL24* | MTOR | **CISH** | IL22RA2* |
| 3 | **PIM1** | 1 | IL24* | PTPN2 | MYC | HRAS | GFAP | AKT3* | AKT3* | **FHL1** |
| 4 | **MCL1** | 1 | MYC | PTPN6 | PTPN11 | CCND1* | CDKN1A | IRF9 | PIAS3* | BCL2L1 |
| 5 | **CISH** | 1 | RAF1 | CREBBP* | RAF1 | EGF* | **PIM1** | **STAT1*** | JAK1* | CREBBP* |
| 6 | SOCS4* | 2 | SOCS4* | RAF1 | PTPN2 | GRB2 | MYC | **PIM1** | EGF* | IRF9 |
| 7 | CCND1* | 1 | **PIM1** | MYC | PTPN6 | MTOR | AKT3* | JAK1* | GFAP | **STAT1*** |
| 8 | AOX1 | 1 | BCL2L1 | PTPN11 | CREBBP* | PTPN11 | JAK1* | IL22RA2* | PIK3CA* | AOX1 |
| 9 | CREBBP* | 1 | SOS1* | IL22RA2* | SOCS4* | RAF1 | PIK3CA* | IL24* | CDKN1A | RAF1 |
| 10 | IRF9 | 1 | PTPN6 | **FHL1** | BCL2L1 | AKT3* | PTPN11 | **MCL1** | STAM2* | CNTF* |
| 11 | BCL2 | 1 | CREBBP* | MTOR | **PIM1** | SOS1* | PTPN2 | MYC | IRF9 | PIAS3* |
| 12 | PTPN2 | 2 | CNTF* | **CISH** | **MCL1** | **STAT1*** | RAF1 | CREBBP* | SOCS4* | CCND1* |
| 13 | JAK1* | 8 | PTPN2 | IRF9 | AKT3* | CREBBP* | BCL2L1 | **FHL1** | **PIM1** | HRAS |
| 14 | MYC | 1 | AKT3* | AKT3* | CNTF* | MYC | CREBBP* | SOCS4* | BCL2 | GRB2 |
| 15 | CDKN1A | 1 | **MCL1** | BCL2L1 | SOS1* | PTPN2 | SOCS4* | PIK3CA* | PTPN6 | PTPN6 |
| 16 | CNTF* | 1 | EGF* | CCND1* | **STAT1*** | STAM2* | **MCL1** | BCL2 | CREBBP* | PTPN2 |
| 17 | STAM2* | 1 | STAM2* | CNTF* | STAM2* | **CISH** | MTOR | CCND1* | IL24* | MYC |
| 18 | HRAS | 2 | IRF9 | PIK3CA* | EGF* | BCL2 | SOS1* | GFAP | SOS1* | **CISH** |
| 19 | MTOR | 1 | **STAT1*** | GRB2 | IRF9 | SOCS4* | HRAS | STAM2* | MYC | CDKN1A |
| 20 | SOS1* | 3 | BCL2 | EGF* | **CISH** | JAK1* | GRB2 | **CISH** | HRAS | BCL2 |
| 21 | IL22RA2* | 4 | **CISH** | BCL2 | BCL2 | BCL2L1 | STAM2* | CNTF* | **STAT1*** | PTPN11 |
| 22 | PIK3CA* | 2 | MTOR | PIAS3* | MTOR | IL22RA2* | AOX1 | AOX1 | IL22RA2* | IL24* |
| 23 | PTPN6 | 1 | JAK1* | SOCS4* | JAK1* | PIAS3* | EGF* | EGF* | RAF1 | GFAP |
| 24 | EGF* | 1 | **FHL1** | STAM2* | **FHL1** | IL24* | IRF9 | HRAS | PTPN2 | SOS1* |
| 25 | GRB2 | 2 | GRB2 | **PIM1** | CCND1* | **FHL1** | BCL2 | GRB2 | **FHL1** | JAK1* |
| 26 | AKT3* | 2 | PIK3CA* | HRAS | GFAP | PTPN6 | CCND1* | BCL2L1 | CNTF* | **PIM1** |
| 27 | IL24* | 1 | CCND1* | AOX1 | PIK3CA* | GFAP | **CISH** | PTPN11 | **MCL1** | PIK3CA* |
| 28 | PTPN11 | 3 | GFAP | SOS1* | GRB2 | **PIM1** | PTPN6 | RAF1 | BCL2L1 | STAM2* |
| 29 | RAF1 | 1 | IL22RA2* | **STAT1*** | IL22RA2* | IRF9 | IL22RA2* | PIAS3* | PTPN11 | AKT3* |
| 30 | PIAS3* | 1 | PIAS3* | JAK1* | AOX1 | CNTF* | **STAT1*** | PTPN6 | GRB2 | **MCL1** |
| 31 | GFAP | 1 | AOX1 | **MCL1** | PIAS3* | **MCL1** | **FHL1** | SOS1* | MTOR | EGF* |
| 32 | BCL2L1 | 1 | HRAS | GFAP | HRAS | AOX1 | PIAS3* | CDKN1A | CCND1* | SOCS4* |

**Table S5:** Ranking of gene nodes in mTor pathway by different methods for the ovarian cancer study.

|  | NetworkHub | degree | t_test | shrinkage_cat | shrinkage_t | Endeavour | PINTA | NGP_ND | NGP_NR | Lin |
| --- | --- | --- | --- | --- | --- | --- | --- | --- | --- | --- |
| 1 | **GSK3B** | 9 | MLST8 | MAP2K1* | MLST8 | TNF | FZD10* | PTEN | RRAGB* | NPRL2* |
| 2 | **IKBKB** | 9 | MAP2K1* | MLST8 | MAP2K1* | PIK3CA* | SLC7A5 | RHEB | MIOS* | **MTOR** |
| 3 | **AKT3*** | 11 | TELO2 | SLC3A2 | SLC3A2 | PTEN | SLC3A2 | **IKBKB** | DVL1* | IRS1 |
| 4 | **MTOR** | 7 | NPRL2* | RPS6KB1* | RPS6KB1* | **GSK3B** | EIF4EBP1 | HRAS* | RHOA | SLC3A2 |
| 5 | **TTI1** | 2 | SLC3A2 | FNIP2* | TELO2 | HRAS* | **AKT3*** | EIF4E* | RPS6 | CLIP1 |
| 6 | MLST8 | 1 | RPS6KB1* | **TTI1** | CLIP1 | MAPK1* | DVL1* | IGF1R* | MAPKAP1 | IGF1* |
| 7 | DEPTOR | 1 | FNIP2* | TNF | NPRL2* | **MTOR** | FLCN | LAMTOR5* | TSC1* | RPS6KA6* |
| 8 | PTEN | 2 | TNF | DEPTOR | FNIP2* | **AKT3*** | MAP2K1* | TSC1* | RRAGD* | TNF |
| 9 | FZD10* | 3 | CLIP1 | CLIP1 | DEPTOR | GRB2 | FNIP2* | DVL1* | EIF4B | DEPTOR |
| 10 | EIF4B | 1 | DEPTOR | IRS1 | TNF | RAF1* | LRP6* | MAPKAP1 | LRP6* | LPIN1 |
| 11 | DVL1* | 3 | **TTI1** | RRAGD* | **TTI1** | IRS1 | PIK3CA* | SLC7A5 | RPS6KB1* | CHUK |
| 12 | TELO2 | 2 | PIK3CA* | PDPK1 | PIK3CA* | SOS1* | RHEB | EIF4EBP1 | IGF1* | PIK3CA* |
| 13 | TSC1* | 8 | CHUK | SGK1 | IRS1 | RPS6KB1* | DDIT4 | STRADB* | PRKCA* | RPS6 |
| 14 | CAB39* | 2 | SGK1 | FZD10* | CHUK | **IKBKB** | LPIN1 | PDPK1 | SGK1 | **GSK3B** |
| 15 | STK11 | 3 | IRS1 | IGF1* | RRAGD* | STK11 | **MTOR** | MAPK1* | PIK3CA* | **AKT3*** |
| 16 | LRP6* | 3 | PDPK1 | EIF4EBP1 | IGF1* | CHUK | EIF4E* | SLC3A2 | GRB2 | TSC1* |
| 17 | RPS6KB1* | 3 | RRAGD* | STK11 | PDPK1 | PRKAA1* | **GSK3B** | ULK1* | FNIP2* | PTEN |
| 18 | RPS6KA6* | 8 | IGF1* | NPRL2* | SGK1 | RPS6KA6* | CLIP1 | FNIP2* | NPRL2* | STK11 |
| 19 | HRAS* | 2 | LAMTOR5* | ATP6V1G3* | LAMTOR5* | EIF4E* | RPS6KB1* | RRAGD* | FZD10* | MLST8 |
| 20 | IGF1R* | 4 | **GSK3B** | CHUK | RPS6 | MAP2K1* | LAMTOR5* | CAB39* | CHUK | LRP6* |
| 21 | STRADB* | 2 | RPS6 | PIK3CA* | FZD10* | TNFRSF1A | ULK1* | EIF4B | PRKAA1* | GRB10 |
| 22 | GRB2 | 2 | FZD10* | LAMTOR5* | ULK1* | IGF1R* | CAB39* | LPIN1 | EIF4E* | ATP6V1G3* |
| 23 | PIK3CA* | 3 | ULK1* | TELO2 | **GSK3B** | LRP6* | PRKCA* | RRAGB* | IRS1 | EIF4B |
| 24 | TNF | 1 | **MTOR** | RPS6 | **MTOR** | WNT16* | RAF1* | RAF1* | TNFRSF1A | IGF1R* |
| 25 | CHUK | 1 | MAPK1* | MAPK1* | MAPK1* | CAB39* | RHOA | FZD10* | RAF1* | HRAS* |
| 26 | IRS1 | 3 | EIF4EBP1 | LPIN1 | EIF4EBP1 | PRR5 | CHUK | IRS1 | FLCN | TELO2 |
| 27 | PRR5 | 2 | CAB39* | LRP6* | GRB10 | TSC1* | TNFRSF1A | MLST8 | SOS1* | RRAGD* |
| 28 | EIF4EBP1 | 1 | GRB10 | **IKBKB** | DDIT4 | MLST8 | PRKAA1* | GRB2 | SLC7A5 | MIOS* |
| 29 | EIF4E* | 1 | DDIT4 | DVL1* | CAB39* | IGF1* | TSC1* | DEPTOR | DEPTOR | RHEB |
| 30 | TNFRSF1A | 2 | PRR5 | SKP2 | RPS6KA6* | DEPTOR | MAPK1* | **MTOR** | PDPK1 | ULK1* |
| 31 | MAPK1* | 9 | RPS6KA6* | DDIT4 | SOS1* | FZD10* | RPS6 | TELO2 | SKP2 | TNFRSF1A |
| 32 | RRAGD* | 3 | SOS1* | SOS1* | PRR5 | STRADB* | EIF4B | PRKAA1* | TNF | LAMTOR5* |
| 33 | SOS1* | 2 | STRADB* | EIF4E* | STRADB* | TELO2 | IGF1R* | DDIT4 | PRR5 | EIF4E* |
| 34 | MAP2K1* | 2 | SKP2 | EIF4B | SKP2 | **TTI1** | **IKBKB** | MAP2K1* | **AKT3*** | **TTI1** |
| 35 | IGF1* | 1 | STK11 | HRAS* | STK11 | RRAGB* | SOS1* | TNF | SLC3A2 | FNIP2* |
| 36 | WNT16* | 2 | EIF4B | **MTOR** | EIF4B | EIF4B | STK11 | SKP2 | **GSK3B** | EIF4EBP1 |
| 37 | RRAGB* | 4 | ATP6V1G3* | **GSK3B** | **IKBKB** | EIF4EBP1 | **TTI1** | RHOA | **IKBKB** | **IKBKB** |
| 38 | RAF1* | 2 | PRKCA* | TSC1* | ATP6V1G3* | FNIP2* | NPRL2* | **GSK3B** | ATP6V1G3* | WNT16* |
| 39 | PRKAA1* | 11 | PTEN | PRR5 | PTEN | RRAGD* | PDPK1 | RPS6 | RPS6KA6* | STRADB* |
| 40 | LAMTOR5* | 2 | RHOA | MAPKAP1 | PRKCA* | DVL1* | PTEN | MIOS* | STRADB* | CAB39* |
| 41 | FNIP2* | 2 | **IKBKB** | IGF1R* | LRP6* | LAMTOR5* | HRAS* | **TTI1** | CAB39* | MAPKAP1 |
| 42 | DDIT4 | 8 | SLC7A5 | PRKAA1* | RHOA | DDIT4 | MIOS* | PRKCA* | MAPK1* | RRAGB* |
| 43 | NPRL2* | 2 | LRP6* | GRB10 | SLC7A5 | NPRL2* | GRB2 | NPRL2* | IGF1R* | RPS6KB1* |
| 44 | RPS6 | 1 | DVL1* | ULK1* | **AKT3*** | RPS6 | IRS1 | GRB10 | LAMTOR5* | MAP2K1* |
| 45 | SKP2 | 1 | FLCN | MIOS* | DVL1* | SKP2 | RPS6KA6* | SOS1* | **TTI1** | MAPK1* |
| 46 | MIOS* | 1 | **AKT3*** | **AKT3*** | FLCN | MIOS* | TELO2 | RPS6KB1* | **MTOR** | FZD10* |
| 47 | PDPK1 | 3 | PRKAA1* | RAF1* | HRAS* | PDPK1 | DEPTOR | IGF1* | DDIT4 | PRKCA* |
| 48 | RHEB | 8 | HRAS* | FLCN | PRKAA1* | RHEB | PRR5 | LRP6* | CLIP1 | PRR5 |
| 49 | MAPKAP1 | 2 | IGF1R* | WNT16* | IGF1R* | MAPKAP1 | SKP2 | PIK3CA* | MAP2K1* | SKP2 |
| 50 | GRB10 | 1 | RRAGB* | RHOA | RRAGB* | GRB10 | IGF1* | ATP6V1G3* | GRB10 | SLC7A5 |
| 51 | ATP6V1G3* | 1 | WNT16* | PTEN | WNT16* | ATP6V1G3* | GRB10 | PRR5 | WNT16* | RAF1* |
| 52 | FLCN | 2 | RAF1* | RRAGB* | RAF1* | FLCN | MAPKAP1 | CHUK | EIF4EBP1 | GRB2 |
| 53 | SLC7A5 | 1 | TSC1* | PRKCA* | EIF4E* | SLC7A5 | MLST8 | RPS6KA6* | TELO2 | FLCN |
| 54 | SLC3A2 | 1 | EIF4E* | TNFRSF1A | TSC1* | SLC3A2 | RRAGD* | FLCN | RHEB | DDIT4 |
| 55 | LPIN1 | 0 | LPIN1 | GRB2 | LPIN1 | LPIN1 | SGK1 | STK11 | ULK1* | SOS1* |
| 56 | RHOA | 0 | MAPKAP1 | RHEB | MAPKAP1 | RHOA | TNF | SGK1 | PTEN | DVL1* |
| 57 | PRKCA* | 0 | GRB2 | CAB39* | GRB2 | PRKCA* | STRADB* | WNT16* | LPIN1 | SGK1 |
| 58 | SGK1 | 0 | RHEB | SLC7A5 | RHEB | SGK1 | WNT16* | TNFRSF1A | HRAS* | RHOA |
| 59 | ULK1* | 0 | MIOS* | RPS6KA6* | MIOS* | ULK1* | RRAGB* | CLIP1 | STK11 | PRKAA1* |
| 60 | CLIP1 | 0 | TNFRSF1A | STRADB* | TNFRSF1A | CLIP1 | ATP6V1G3* | **AKT3*** | MLST8 | PDPK1 |
