# Supplementary material for: Network hub-node prioritization of gene regulation with intra-network association
Source: BMC Bioinformatics. 2020 Mar 12;21:101. doi: 10.1186/s12859-020-3444-7 (PMC7069025; doi:10.1186/s12859-020-3444-7)
Supplement: Supplementary file 2 — Additional file 2: Figure S1. Boxplots of local weights (left column) and topology weights (right column). Here the thresholds are set at conservative values: 0.05 for αL, 1 for αS, and no limit for D so that all nodes are included. Figure S2: Analyses of the mTor pathway for the breast cancer study. Figure S3: Analyses of the estrogen pathway for the breast cancer study. Figure S4: Analyses of the JAK-STAT pathway for the breast cancer study. Figure S5: The hub ranking rate of NetworkHub corresponding to different threshold values before and after the pathway association test, under four scenarios. A: Before. B: After. [file 12859_2020_3444_MOESM2_ESM.pdf]

Supplementary Figures

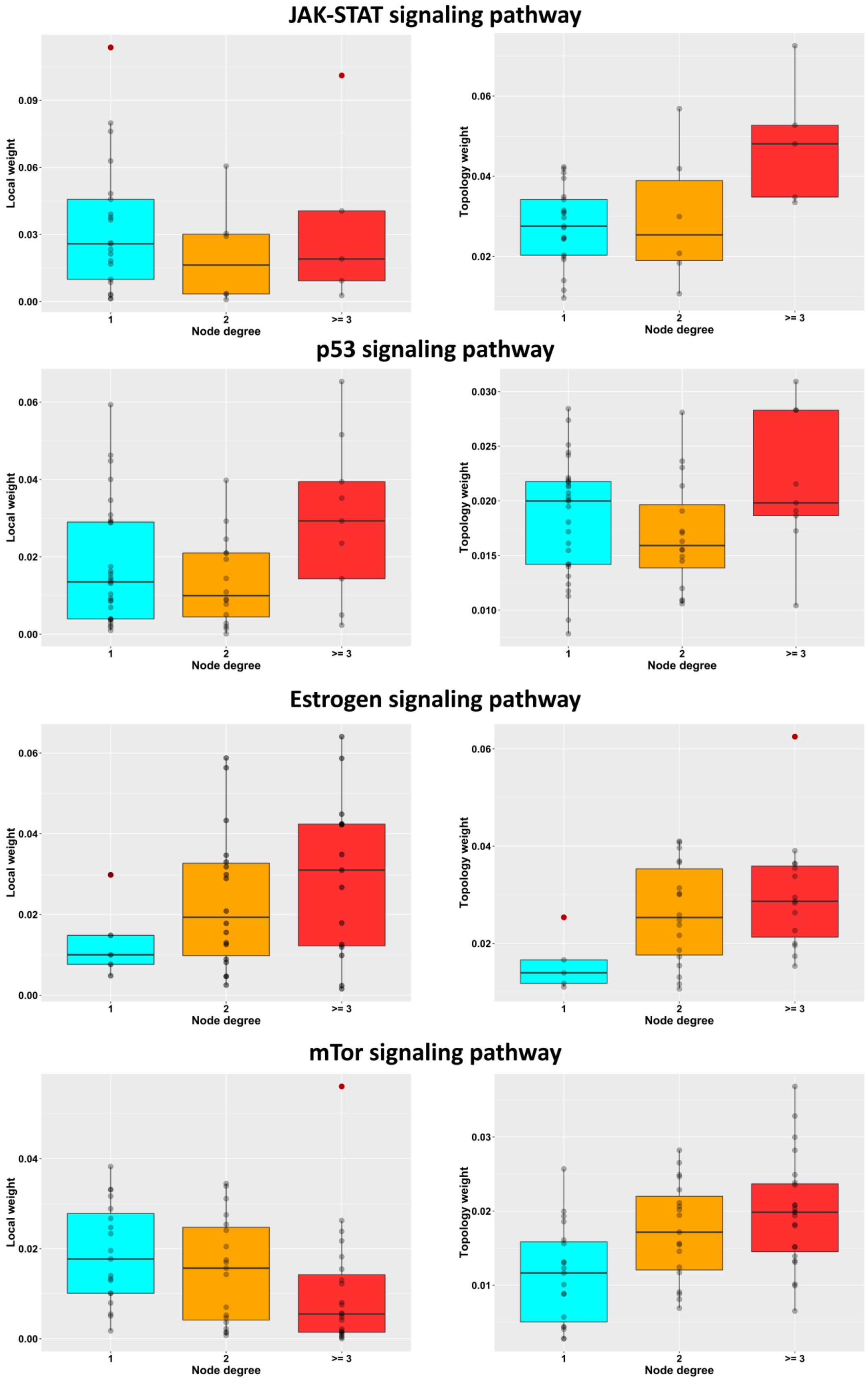

Figure S1. Boxplots of local weights (left column) and topology weights (right column). Here the thresholds are set at conservative values: 0.05 for α_L, 1 for α_S, and no limit for D so that all nodes are included.

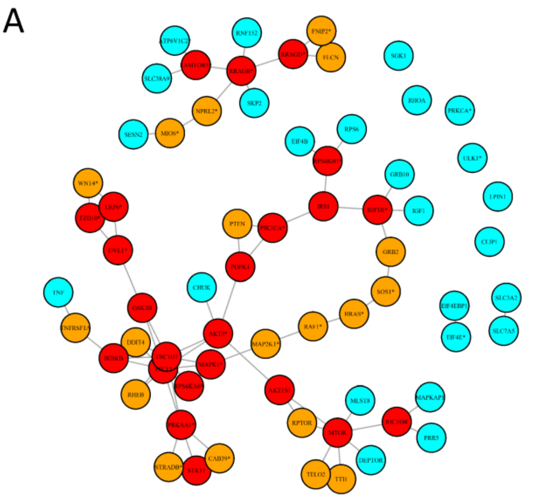

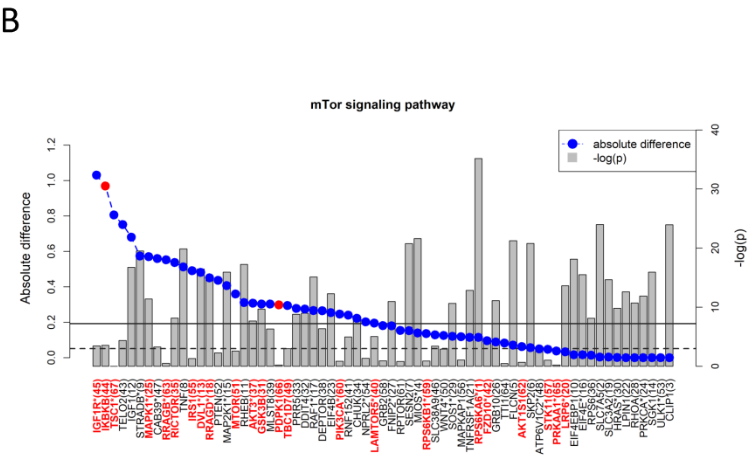

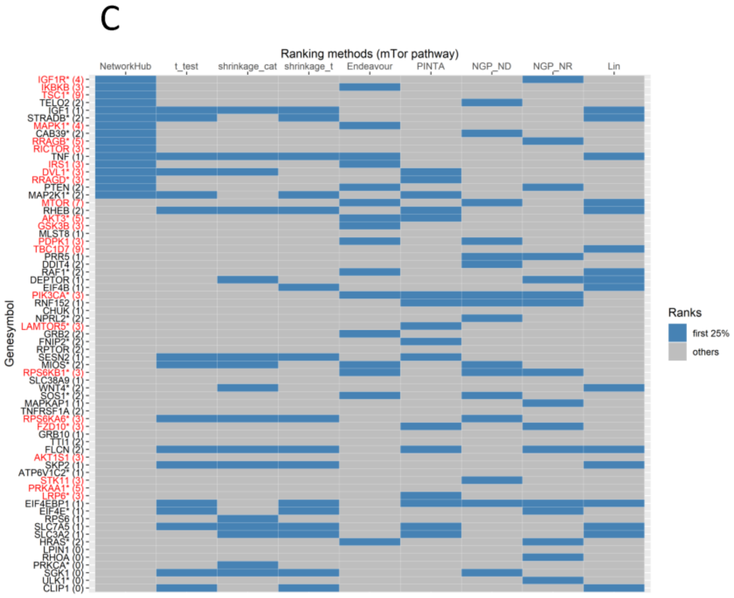

D

rank	NetworkHub	degree	t_test	shrinkage_cat	shrinkage_t	Endeavour	PINTA	NGP_ND	NGP_NR	Lin
1	**IGF1R***	4	RPS6KA6*	SESN2	TNF	PIK3CA*	LAMTOR5*	MIOS*	DEPTOR	**IGF1**
2	**IKBKB**	3	SLC7A5	TNF	SESN2	PTEN	FZD10*	STK11	RPS6KB1*	MTOR
3	**TSC1***	9	CLIP1	RPS6KA6*	SLC3A2	TNF	SESN2	RPS6KB1*	FZD10*	EIF4EBP1
4	**TELO2**	2	MIOS*	SKP2	SKP2	GSK3B	SLC7A5	PIK3CA*	PRR5	FLCN
5	**IGF1**	1	FLCN	**IGF1**	RPS6KA6*	HRAS*	RRAGD*	MTOR	FLCN	DEPTOR
6	STRADB*	2	SKP2	SLC3A2	SLC7A5	MAPK1*	EIF4EBP1	**TELO2**	MAPKAP1	SLC3A2
7	MAPK1*	4	SESN2	DVL1*	MAP2K1*	PDPK1	SLC3A2	SOS1*	PIK3CA*	WNT4*
8	CAB39*	2	TNF	SLC7A5	STRADB*	MTOR	AKT3*	DDIT4	RNF152	STRADB*
9	RRAGB*	5	STRADB*	FLCN	FLCN	GRB2	DVL1*	CAB39*	EIF4EBP1	SKP2
10	RICTOR	3	EIF4EBP1	SGK1	RHEB	AKT3*	RNF152	RNF152	EIF4E*	TNF
11	TNF	1	RHEB	MIOS*	**IGF1**	IRS1	FLCN	RPS6KA6*	**IGF1R***	CLIP1
12	IRS1	3	**IGF1**	RHEB	CLIP1	**IKBKB**	MAP2K1*	EIF4EBP1	ULK1*	RAF1*
13	DVL1*	3	DVL1*	PRKCA*	SGK1	RPS6KB1*	RHEB	PDPK1	RRAGB*	SLC7A5
14	RRAGD*	3	SGK1	DEPTOR	EIF4B	RAF1*	FNIP2*	PRR5	RHOA	TBC1D7
15	PTEN	2	MAP2K1*	WNT4*	EIF4E*	MIOS*	PIK3CA*	SGK1	HRAS*	RHEB
16	MAP2K1*	2	EIF4E*	RPS6	EIF4EBP1	SOS1*	LRP6*	NPRL2*	PTEN	EIF4B
17	MTOR	7	RAF1*	CHUK	MIOS*	STK11	DDIT4	FLCN	LAMTOR5*	RPS6
18	RHEB	2	RRAGD*	HRAS*	RRAGD*	PRKAA1*	MTOR	PTEN	MAPK1*	FZD10*
19	AKT3*	5	SLC3A2	DDIT4	RHOA	AKT1S1	RICTOR	LAMTOR5*	CHUK	RPS6KB1*
20	GSK3B	3	LRP6*	EIF4EBP1	LPIN1	CHUK	LPIN1	RHEB	AKT3*	SESN2

Figure S2: Analyses of the mTor pathway for the breast cancer study.

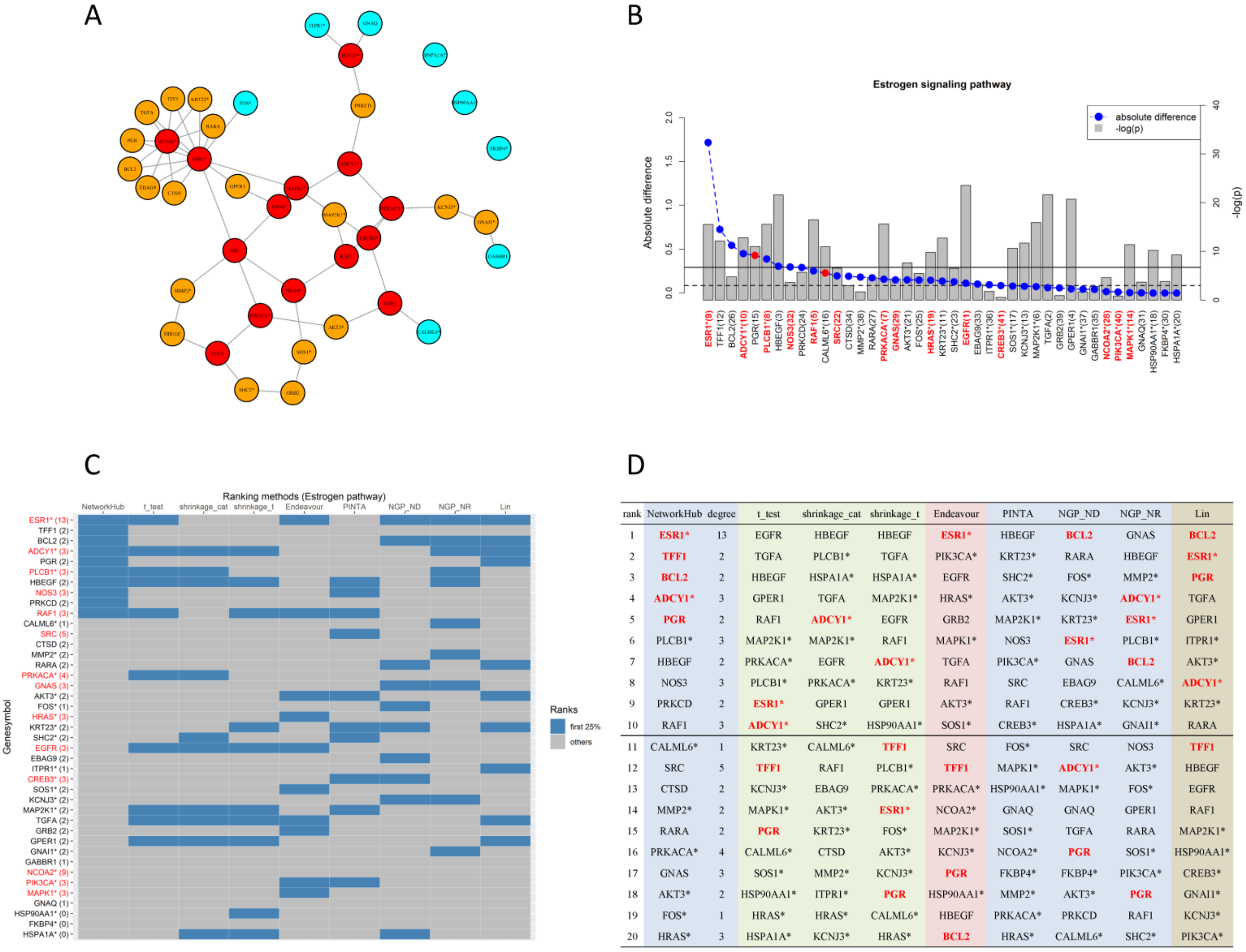

rank	NetworkHub	degree	t_test	shrinkage_cat	shrinkage_t	Endeavour	PINTA	NGP_ND	NGP_NR	Lin
1	**ESR1***	13	EGFR	HBEGF	HBEGF	**ESR1***	HBEGF	**BCL2**	GNAS	**BCL2**
2	**TFF1**	2	TGFA	PLCB1*	TGFA	PIK3CA*	KRT23*	RARA	HBEGF	**ESR1***
3	**BCL2**	2	HBEGF	HSPA1A*	HSPA1A*	EGFR	SHC2*	FOS*	MMP2*	**PGR**
4	**ADCY1***	3	GPER1	TGFA	MAP2K1*	HRAS*	AKT3*	KCNJ3*	**ADCY1***	TGFA
5	**PGR**	2	RAF1	**ADCY1***	EGFR	GRB2	MAP2K1*	KRT23*	**ESR1***	GPER1
6	PLCB1*	3	MAP2K1*	MAP2K1*	RAF1	MAPK1*	NOS3	**ESR1***	PLCB1*	ITPR1*
7	HBEGF	2	PRKACA*	EGFR	**ADCY1***	TGFA	PIK3CA*	GNAS	**BCL2**	AKT3*
8	NOS3	3	PLCB1*	PRKACA*	KRT23*	RAF1	SRC	EBAG9	CALML6*	**ADCY1***
9	PRKCD	2	**ESR1***	GPER1	GPER1	AKT3*	RAF1	CREB3*	KCNJ3*	KRT23*
10	RAF1	3	**ADCY1***	SHC2*	HSP90AA1*	SOS1*	CREB3*	HSPA1A*	GNAI1*	RARA
11	CALML6*	1	KRT23*	CALML6*	**TFF1**	SRC	FOS*	SRC	NOS3	**TFF1**
12	SRC	5	**TFF1**	RAF1	PLCB1*	**TFF1**	MAPK1*	**ADCY1***	AKT3*	HBEGF
13	CTSD	2	KCNJ3*	EBAG9	PRKACA*	PRKACA*	HSP90AA1*	MAPK1*	FOS*	EGFR
14	MMP2*	2	MAPK1*	AKT3*	**ESR1***	NCOA2*	GNAQ	GNAQ	GPER1	RAF1
15	RARA	2	**PGR**	KRT23*	FOS*	MAP2K1*	SOS1*	TGFA	RARA	MAP2K1*
16	PRKACA*	4	CALML6*	CTSD	AKT3*	KCNJ3*	NCOA2*	**PGR**	SOS1*	HSP90AA1*
17	GNAS	3	SOS1*	MMP2*	KCNJ3*	**PGR**	FKBP4*	FKBP4*	PIK3CA*	CREB3*
18	AKT3*	2	HSP90AA1*	ITPR1*	**PGR**	HSP90AA1*	MMP2*	AKT3*	**PGR**	GNAI1*
19	FOS*	1	HRAS*	HRAS*	CALML6*	HBEGF	PRKACA*	PRKCD	RAF1	KCNJ3*
20	HRAS*	3	HSPA1A*	KCNJ3*	HRAS*	**BCL2**	HRAS*	CALML6*	SHC2*	PIK3CA*

Figure S3: Analyses of the estrogen pathway for the breast cancer study.

A

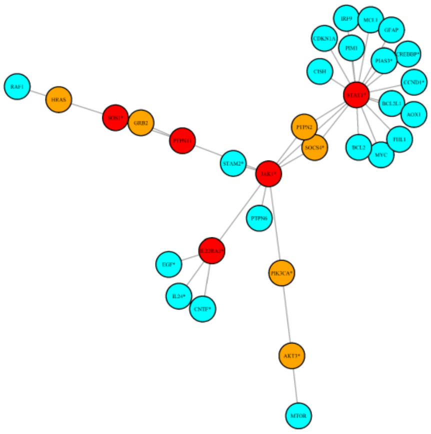

B

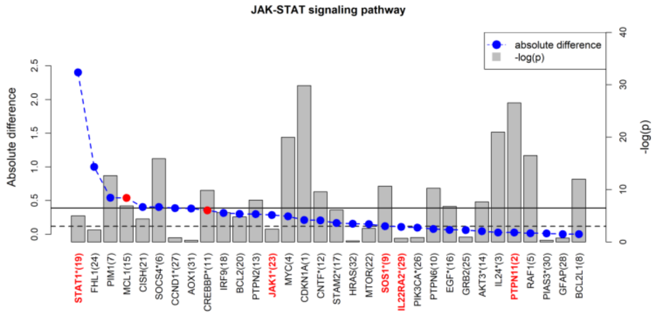

C

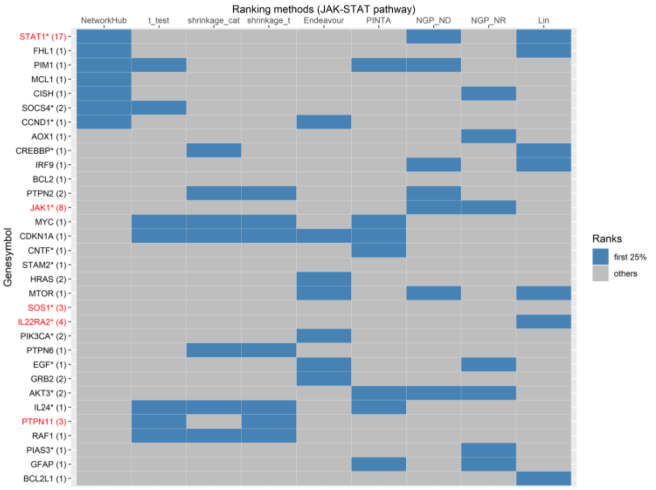

D

rank	NetworkHub	degree	t_test	shrinkage_cat	shrinkage_t	Endeavour	PINTA	NGP_ND	NGP_NR	Lin
1	**STAT1***	17	CDKN1A	IL24*	IL24*	PIK3CA*	CNTF*	PTPN2	AOX1	MTOR
2	**FHL1**	1	PTPN11	CDKN1A	CDKN1A	CDKN1A	IL24*	MTOR	**CISH**	IL22RA2*
3	**PIM1**	1	IL24*	PTPN2	MYC	HRAS	GFAP	AKT3*	AKT3*	**FHL1**
4	**MCL1**	1	MYC	PTPN6	PTPN11	CCND1*	CDKN1A	IRF9	PIAS3*	BCL2L1
5	**CISH**	1	RAF1	CREBBP*	RAF1	EGF*	**PIM1**	**STAT1***	JAK1*	CREBBP*
6	SOCS4*	2	SOCS4*	RAF1	PTPN2	GRB2	MYC	**PIM1**	EGF*	IRF9
7	CCND1*	1	**PIM1**	MYC	PTPN6	MTOR	AKT3*	JAK1*	GFAP	**STAT1***
8	AOX1	1	BCL2L1	PTPN11	CREBBP*	PTPN11	JAK1*	IL22RA2*	PIK3CA*	AOX1
9	CREBBP*	1	SOS1*	IL22RA2*	SOCS4*	RAF1	PIK3CA*	IL24*	CDKN1A	RAF1
10	IRF9	1	PTPN6	**FHL1**	BCL2L1	AKT3*	PTPN11	**MCL1**	STAM2*	CNTF*
11	BCL2	1	CREBBP*	MTOR	**PIM1**	SOS1*	PTPN2	MYC	IRF9	PIAS3*
12	PTPN2	2	CNTF*	**CISH**	**MCL1**	**STAT1***	RAF1	CREBBP*	SOCS4*	CCND1*
13	JAK1*	8	PTPN2	IRF9	AKT3*	CREBBP*	BCL2L1	**FHL1**	**PIM1**	HRAS
14	MYC	1	AKT3*	AKT3*	CNTF*	MYC	CREBBP*	SOCS4*	BCL2	GRB2
15	CDKN1A	1	**MCL1**	BCL2L1	SOS1*	PTPN2	SOCS4*	PIK3CA*	PTPN6	PTPN6
16	CNTF*	1	EGF*	CCND1*	**STAT1***	STAM2*	**MCL1**	BCL2	CREBBP*	PTPN2
17	STAM2*	1	STAM2*	CNTF*	STAM2*	**CISH**	MTOR	CCND1*	IL24*	MYC
18	HRAS	2	IRF9	PIK3CA*	EGF*	BCL2	SOS1*	GFAP	SOS1*	**CISH**
19	MTOR	1	**STAT1***	GRB2	IRF9	SOCS4*	HRAS	STAM2*	MYC	CDKN1A
20	SOS1*	3	BCL2	EGF*	**CISH**	JAK1*	GRB2	**CISH**	HRAS	BCL2

Figure S4: Analyses of the JAK-STAT pathway for the breast cancer study.

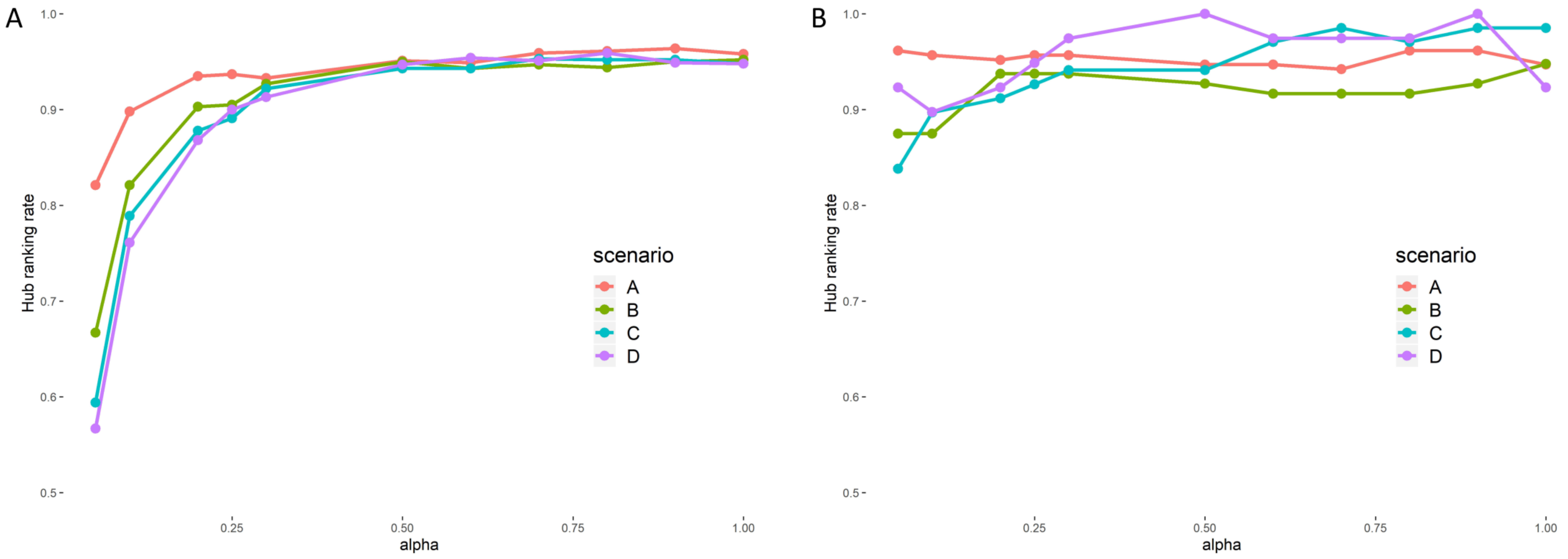

Figure S5: The hub ranking rate of NetworkHub corresponding to different threshold values before and after the pathway association test, under four scenarios. **A:** Before. **B:** After.
